# *De-Novo* Learning of Genome-Scale Regulatory Networks in *S. cerevisiae*

**DOI:** 10.1371/journal.pone.0106479

**Published:** 2014-09-12

**Authors:** Sisi Ma, Patrick Kemmeren, David Gresham, Alexander Statnikov

**Affiliations:** 1 Center for Health Informatics and Bioinformatics, New York University Langone Medical Center, New York, NY, United States of America; 2 Department of Medicine, New York University School of Medicine, New York, NY, United States of America; 3 Molecular Cancer Research, Center for Molecular Medicine, University Medical Center, Utrecht, The Netherlands; 4 Department of Biology, New York University, New York, NY, United States of America; Leibniz-Institute for Farm Animal Biology (FBN), Germany

## Abstract

De-novo reverse-engineering of genome-scale regulatory networks is a fundamental problem of biological and translational research. One of the major obstacles in developing and evaluating approaches for de-novo gene network reconstruction is the absence of high-quality genome-scale gold-standard networks of direct regulatory interactions. To establish a foundation for assessing the accuracy of de-novo gene network reverse-engineering, we constructed high-quality genome-scale gold-standard networks of direct regulatory interactions in *Saccharomyces cerevisiae* that incorporate binding and gene knockout data. Then we used 7 performance metrics to assess accuracy of 18 statistical association-based approaches for de-novo network reverse-engineering in 13 different datasets spanning over 4 data types. We found that most reconstructed networks had statistically significant accuracies. We also determined which statistical approaches and datasets/data types lead to networks with better reconstruction accuracies. While we found that de-novo reverse-engineering of the entire network is a challenging problem, it is possible to reconstruct sub-networks around some transcription factors with good accuracy. The latter transcription factors can be identified by assessing their connectivity in the inferred networks. Overall, this study provides the gene network reverse-engineering community with a rigorous assessment of the accuracy of *S. cerevisiae* gene network reconstruction and variability in performance of various approaches for learning both the entire network and sub-networks around transcription factors.

## Introduction

One of the fundamental problems of modern biology is reverse-engineering of genome-scale regulatory networks. Addressing this problem is essential to expanding understanding of normal and pathologic cellular conditions and can lead to development of new drugs and therapies. While there are many databases that store biological pathways (e.g., KEGG and Ingenuity Pathway Analysis), these databases are often inaccurate and/or incomplete because their knowledge is derived from a multitude of biological systems and conditions that may not correspond to the problem at hand. Furthermore, pathways in these databases are affected by variability of the employed computational and experimental methods and their reproducibility characteristics [1]–[3]. Therefore, there is a strong need for reverse-engineering of genome-scale regulatory networks *de novo* from data.

**Figure 1:**
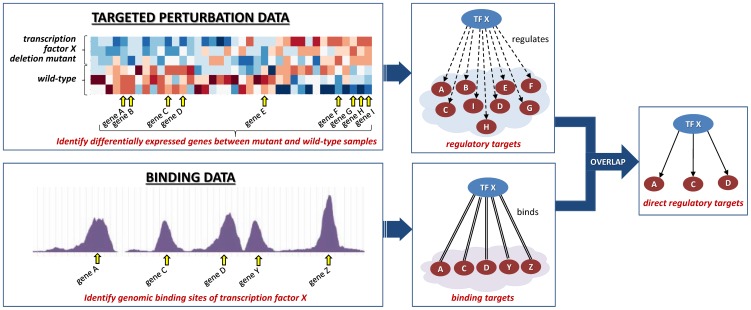
Construction of gene regulatory networks by integrating targeted perturbation data with binding data. The relations in constructed gene regulatory network correspond to direct regulatory interactions.

Gene regulatory networks can be constructed by integrating targeted perturbation data (e.g., gene knockouts or overexpression of transcription factors) with binding data (e.g., chromatin immunoprecipitation) (**Figure 1**). By knocking-out/deleting or over-expressing transcription factor X and comparing the expression level of other genes with the wild-type strain, one can determine *regulatory targets* of X. On the other hand, a binding assay allows identification of the *binding targets* of X. The overlap of regulatory targets and binding targets defines the set of *direct regulatory targets* of X which are graphically represented in gene regulatory networks. While modern methods in biology enable performing such studies in a variety of model systems, they are typically expensive to perform on a genome-scale and often unfeasible in humans.

However, the wide-spread use of genomic profiling technologies over the last two decades led to development of thousands of observational, i.e. non-perturbation datasets (e.g., from case-control and case-series studies), that are freely available in public repositories such as GEO [4] and ArrayExpress [5]. In addition, the computational community has recently provided many algorithms that can infer causal relations from non-perturbation data [6]–[10]; some of them have been adopted to accommodate the high dimensionalities of modern genomics data [11], [12], and some methods even lead to Nobel awards in domains outside of biomedicine [13]–[16]. The question is whether these computational methodologies can accurately learn de-novo gene regulatory networks from highly abundant data in the public domain?

Fortunately, this question has recently received attention in the scientific community [17]–[21]. However, the major obstacle in testing gene network reverse-engineering methods is the absence of high-quality genome-scale gold-standards of direct regulatory interactions that are derived by integrating targeted perturbation with binding data (see **Table 1**). Another problem is that currently the scientific community primarily uses perturbation data for gene network inference (many studies use compendium microarray data that is obtained by merging a large number of studies, predominantly with deletion mutants), while results based on observational data are more important, since the latter data is easier and cheaper to obtain. In general, it is unknown what types of datasets are more suitable for gene network reverse-engineering studies.

**Table 1:** Assessment of currently available genome-scale gold-standard networks used by prior gene network reverse-engineering studies.

Gold-standard	Description	Limitations	Used by
#1	E. Coli network from RegulonDB, a curated database of regulatory interactions obtained through literature search [50]	• Unknown quality	DREAM2 [17], DREAM5 [18], [19], [21]
		• Heterogeneous data sources and experimental methods	
#2	S. Cerevisiae network from binding data [51]	• Binding relations can be non-functional [28]	[20]
		• Higher quality binding data exists [33] and is utilized in gold-standard #3	
#3	S. Cerevisiae network from binding data [33]	• Binding relations can be non-functional [28]	DREAM5 [18], [19], [21]
#4	S. Cerevisiae network from YEASTRACT, a curated database of regulatory interactions obtained through literature search [52], [53]	• Unknown quality	DREAM5 [18]
		• Heterogeneous data sources and experimental methods	
#5	S. Cerevisiae network from deletion mutants [54]	• Inferred transcription relations can be indirect	DREAM5 [18]

To address gaps in prior research, this study focuses on *S. cerevisiae*, one of the most well-studied model organisms with a wide range of available genome-scale data. We first constructed high-quality genome-scale gold-standards of regulatory interaction and then assessed 18 statistical association-based approaches (from both bivariate analysis and multivariate causal graph-based methods) for de-novo network reverse-engineering in 13 different datasets that span over 4 data types: (i) observational data consisting of biological wild-type replicates, (ii) observational data obtained across time and/or environmental conditions, (iii) compendium (semi-perturbation) data, and (iv) perturbation data. This study uses de-novo methods based on statistical association [11], [12], [22]–[24] because they are state-of-the-art [19] and are most prevalent in the community. In the course of this study, the following four questions are addressed: First, how accurately can one infer genome-scale networks with statistical association-based de-novo methods? Second, which datasets/data designs should be used for network inference? Third, which statistical methods lead to better accuracy? Fourth, is it possible to identify sub-networks in the entire network that can be reconstructed with high accuracy? To make conclusions of the study more useful to the community, results for 7 commonly used performance metrics are reported.

## Results

### Gold-standard gene regulatory networks integrate transcription factor-gene binding with perturbation (deletion mutants) data

The analysis of targeted perturbation (deletion mutants) data described in the Methods section resulted in a network with 991,444 regulatory relations involving 5,395 genes, including 118 transcription factors (**Spreadsheet S1**).

The analysis of binding data described in the Methods section resulted in the following three networks: Binding network #1 (most conservative) involves 2,075 genes (including 114 transcription factors) and 4,034 binding relations. Binding network #2 (intermediate) involves 3,113 genes (including 115 transcription factors) and 8,392 binding relations. Binding network #3 (most liberal) involves 3,955 genes (including 116 transcription factors) and 13,050 binding relations. All identified binding interactions are provided in **Spreadsheet S2**.

**Figure 2:**
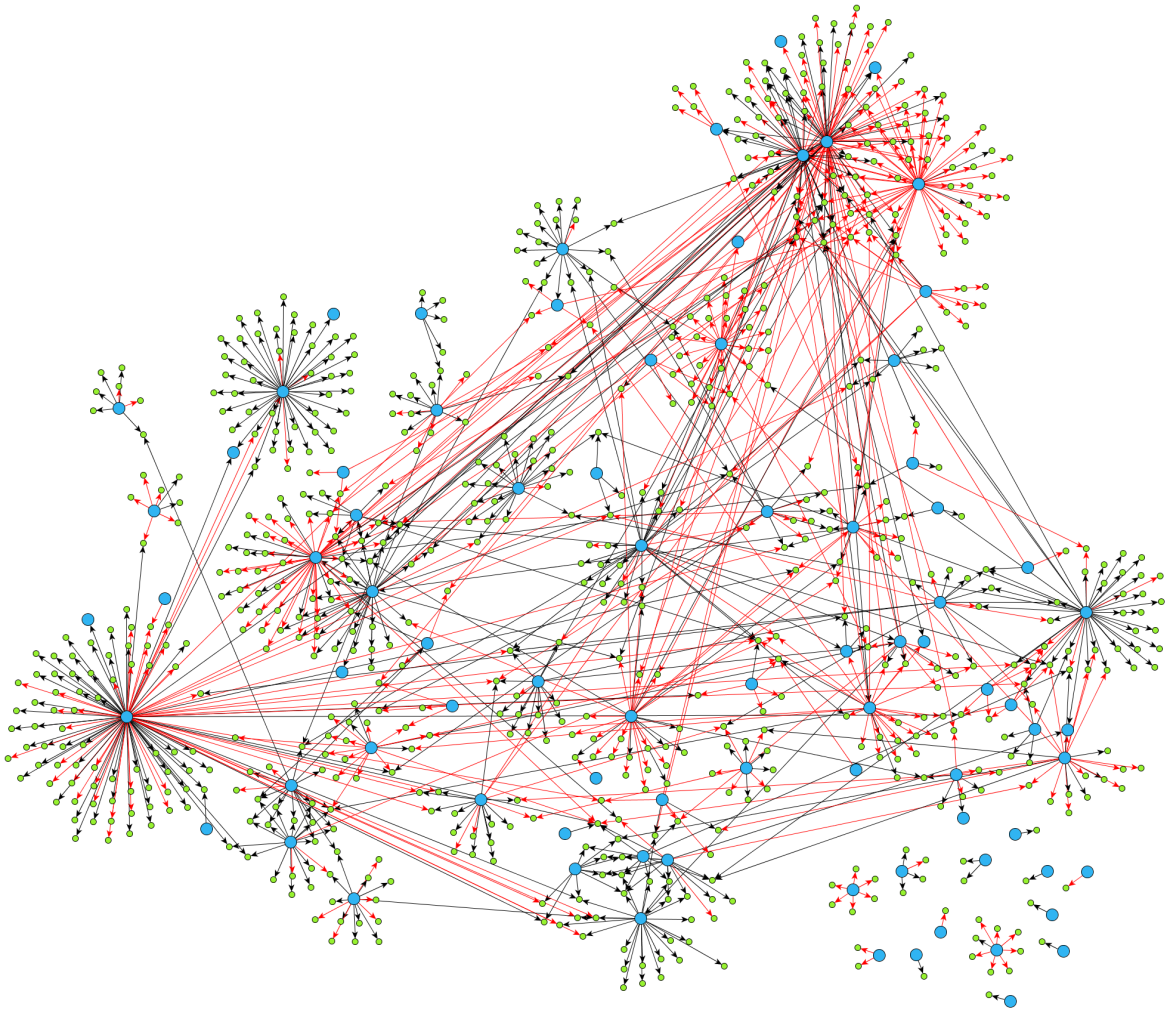
Gold-standard gene regulatory network #1. Transcription factors are shown with large blue circles, and other genes are shown with small green circles. Edges in the network represent direct regulatory interactions. Inhibiting edges are shown with red, and excitatory edges are shown with black.

**Figure 3:**
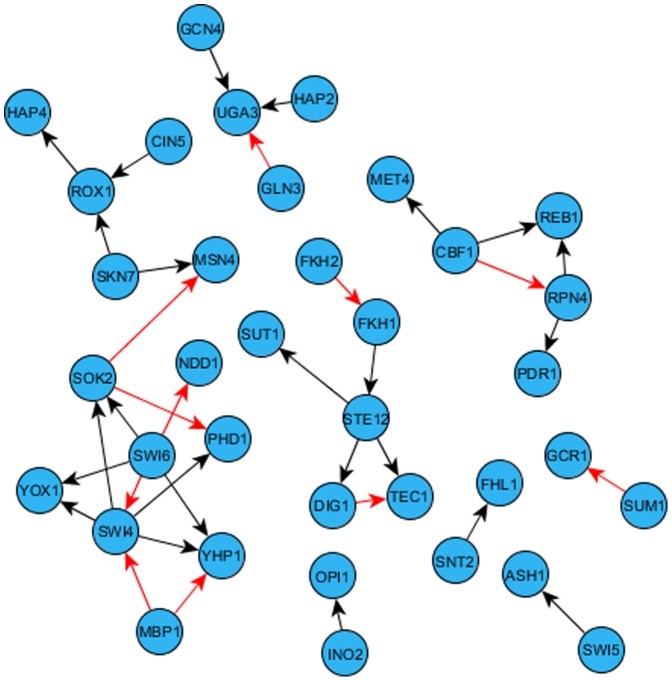
Direct regulatory interactions between transcription factors in gold-standard gene regulatory network #1. Inhibiting edges are shown with red, and excitatory edges are shown with black.

**Figure 4:**
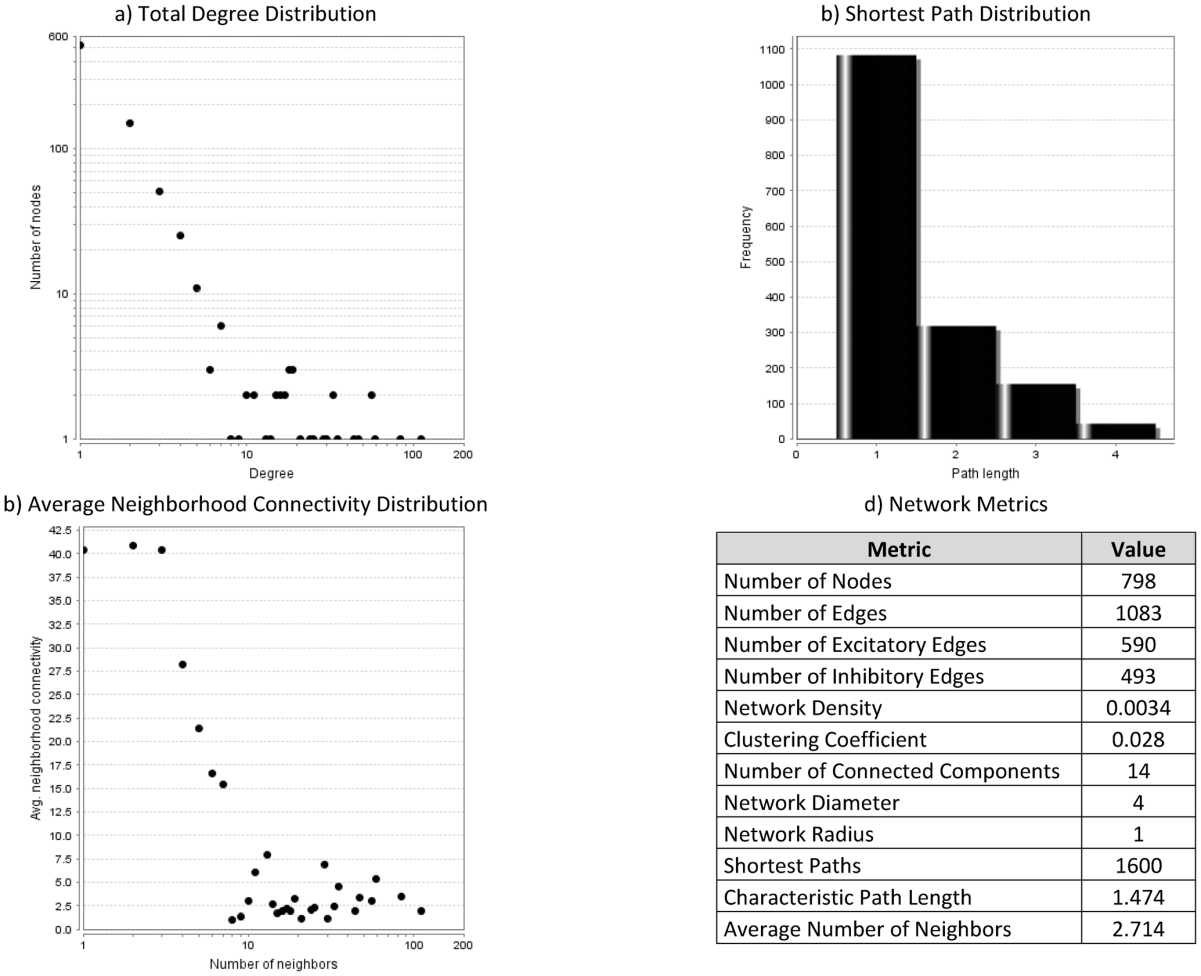
Topological analysis of gold-standard gene regulatory network #1. The analysis was performed in Cytoscape with NetworkAnalyzer.

Integration of binding and perturbation data resulted in three gold-standard networks with direct regulatory interactions (**Table 2**). Identified direct regulatory interactions are listed in **Spreadsheet S3**. **Figures 2** and **3** visualize the gold-standard network #1 for all genes and only transcription factors, respectively. **Figure 4** presents a topological analysis of that gold-standard network. Similar data is provided for gold-standard networks #2 and #3 in **Figures S1–S6**.

**Table 2:** Overlapping identified binding with regulatory relations results in gold-standard networks with direct regulatory relations.

Gold-standard network #	Binding Network			Regulatory network	Gold-standard network (integrating binding and regulatory networks)	
	*Binding threshold*	*Evolutionary conservation of binding sequences* *(# of species)*	*# of edges* *(binding relations)*	*# of edges* *(regulatory relations)*	*# of edges* *(direct regulatory relations)*	*Statistical significance of the overlap* *(p-value from hypergeometric test)*
1	0.001	2	4,034	991,444	1,083	<10^−16^
2	0.005	1	8,392		1,785	<10^−16^
3	0.005	0	13,050		2,403	<10^−16^

### Assessment of the accuracy of network learning with sensitivity and specificity metrics

The network reconstruction results presented below were obtained from the most conservative gold-standard network #1 (**Table 2**). Results from the remaining two gold-standard networks are similar and are provided in **Tables S4–S9**.

**Table 3** provides values of sensitivity and specificity and **Table 4** provides a combined sensitivity/specificity Euclidean distance-based metric (see Methods) for 18 statistical approaches for reverse-engineering applied to 13 datasets, resulting in 234 inferred networks (see **Table S1** for a colored version of **Table 3** and **Table 4**, where color denotes ranking of performances). The best result for combined sensitivity/specificity metric ( = 0.64, corresponding to sensitivity  = 0.52 and specificity  = 0.58) is achieved in Hughes2 dataset by application of bivariate analysis with G2 test and 5% alpha threshold. The best 5% ranking results (see **Table S1** part B) according to the combined metric (12 networks out of 234) correspond to bivariate analysis (10 networks) and GLL with conditioning on one gene (2 networks). In terms of datasets, 4 out of 12 best networks originate from Hughes1, 4 from Hughes2, 2 from GPL90, and 2 from Gasch. There is a large variability in accuracy of statistical approaches averaged over 13 datasets, and the most accurate approaches are bivariate (combined metric  = 0.75–0.77 versus 0.85–0.98 for other methods). The variability in accuracy of datasets averaged over 18 statistical approaches is smaller, and the best results are achieved in Gresham (combined metric  = 0.82), Smith (0.84), and Holstege4 (0.84) datasets (versus 0.85–0.89 for the remaining datasets). If we perform averaging over all statistical approaches and datasets belonging to the same data type, the best accuracy is achieved by observational data due to change in time/environment and by compendium data (combined metric  = 0.86), followed by perturbation data (0.87) and observational data consisting of biological wild-type replicates (0.88).

**Table 3:** Sensitivity and specificity.

			Observational (Biological Replicates)		Observational (Environment/Time)				Semi-experimental (Compendium)		Experimental				
Statistics	Conditioning	Post-Processing	Holstege1	Holstege2	Gresham	Gasch	Smith	Yeung	M3D	GPL90	Hughes1	Hughes2	Hu	Holstege3	Holstege4
Fisher's Z	None	FDR, “AND” rule	0.63|0.37	**0.79|0.22**	**0.58|0.44**	**0.49|0.53**	**0.76|0.28**	0.75|0.19	0.74|0.26	**0.78|0.25**	**0.43|0.65**	**0.35|0.73**	0.33|0.68	0.73|0.25	**0.71|0.33**
Fisher's Z	None	FDR, “OR” rule	0.65|0.35	**0.81|0.20**	**0.62|0.41**	**0.53|0.49**	**0.80|0.25**	0.77|0.18	0.76|0.25	**0.79|0.24**	**0.45|0.61**	**0.38|0.69**	0.36|0.64	0.74|0.24	**0.72|0.31**
Fisher's Z	None	None	0.68|0.32	**0.82|0.20**	**0.65|0.38**	**0.57|0.45**	**0.81|0.23**	0.78|0.17	0.77|0.24	**0.80|0.23**	**0.52|0.54**	**0.47|0.61**	0.43|0.57	0.77|0.22	**0.75|0.29**
Fisher's Z	1 gene	“AND” rule	0.06|0.94	**0.06|0.95**	0.03|0.97	**0.07|0.96**	**0.10|0.95**	**0.11|0.93**	**0.15|0.91**	**0.20|0.85**	**0.11|0.92**	**0.11|0.93**	0.07|0.94	0.12|0.88	**0.14|0.91**
Fisher's Z	1 gene	“OR” rule	**0.07|0.94**	**0.07|0.94**	0.04|0.97	**0.07|0.96**	**0.12|0.94**	**0.11|0.92**	**0.16|0.90**	**0.21|0.84**	**0.12|0.91**	**0.12|0.93**	0.07|0.93	0.13|0.88	**0.15|0.90**
Fisher's Z	2 genes	“AND” rule	0.01|0.99	0.01|0.99	**0.01|1.00**	**0.02|0.99**	**0.03|0.99**	**0.03|0.99**	**0.03|0.98**	**0.06|0.96**	**0.03|0.98**	**0.04|0.98**	0.02|0.98	**0.04|0.98**	**0.03|0.98**
Fisher's Z	2 genes	“OR” rule	0.02|0.98	**0.02|0.98**	**0.02|0.99**	**0.03|0.99**	**0.04|0.98**	**0.05|0.98**	**0.05|0.97**	**0.08|0.95**	**0.04|0.97**	**0.05|0.97**	**0.03|0.98**	**0.05|0.96**	**0.05|0.97**
Fisher's Z	3 genes	“AND” rule	0.01|1.00	0.01|1.00	0.00|1.00	**0.01|1.00**	**0.03|0.99**	**0.02|0.99**	**0.01|0.99**	**0.03|0.98**	**0.02|0.99**	**0.02|0.99**	**0.01|0.99**	**0.02|0.99**	**0.02|0.99**
Fisher's Z	3 genes	“OR” rule	**0.02|0.99**	0.01|0.99	0.01|0.99	**0.02|0.99**	**0.04|0.99**	**0.04|0.98**	**0.04|0.98**	**0.05|0.97**	**0.03|0.98**	**0.04|0.98**	**0.02|0.98**	**0.04|0.98**	**0.04|0.98**
G2	None	FDR, “AND” rule	0.39|0.59	0.62|0.37	0.49|0.50	**0.43|0.59**	**0.71|0.34**	0.80|0.14	**0.90|0.11**	**0.89|0.16**	**0.24|0.83**	**0.32|0.76**	0.21|0.80	0.69|0.30	**0.66|0.38**
G2	None	FDR, “OR” rule	0.44|0.54	0.66|0.33	0.56|0.44	**0.50|0.52**	**0.76|0.28**	0.82|0.12	**0.91|0.11**	**0.90|0.15**	**0.30|0.78**	**0.39|0.69**	0.26|0.74	0.72|0.27	**0.71|0.33**
G2	None	None	0.50|0.49	0.69|0.31	0.60|0.38	**0.55|0.47**	**0.79|0.25**	0.83|0.12	**0.91|0.10**	**0.90|0.14**	**0.43|0.65**	**0.52|0.58**	0.36|0.65	0.76|0.23	**0.76|0.29**
G2	1 gene	“AND” rule	0.04|0.96	0.14|0.86	0.59|0.39	**0.04|0.98**	**0.19|0.87**	**0.25|0.78**	**0.37|0.68**	**0.47|0.59**	**0.02|0.99**	**0.02|0.99**	**0.04|0.98**	0.14|0.85	**0.14|0.90**
G2	1 gene	“OR” rule	0.04|0.95	0.15|0.85	0.60|0.38	**0.06|0.96**	**0.22|0.85**	**0.26|0.77**	**0.39|0.66**	**0.48|0.59**	**0.04|0.97**	**0.04|0.97**	**0.05|0.97**	0.16|0.83	**0.17|0.88**
G2	2 genes	“AND” rule	0.04|0.96	0.14|0.86	0.52|0.47	**0.04|0.98**	**0.19|0.87**	**0.04|0.98**	**0.06|0.97**	**0.15|0.90**	**0.02|0.99**	**0.02|0.99**	**0.04|0.98**	0.02|0.98	**0.14|0.90**
G2	2 genes	“OR” rule	0.04|0.95	0.15|0.85	0.60|0.39	**0.06|0.96**	**0.22|0.85**	**0.06|0.97**	**0.09|0.93**	**0.17|0.87**	**0.04|0.97**	**0.04|0.97**	**0.05|0.97**	0.04|0.96	**0.17|0.88**
G2	3 genes	“AND” rule	0.04|0.96	0.09|0.91	0.09|0.89	**0.03|0.98**	**0.16|0.91**	**0.04|0.98**	**0.06|0.97**	**0.06|0.97**	**0.02|0.99**	**0.02|0.99**	**0.04|0.98**	0.02|0.98	**0.14|0.90**
G2	3 genes	“OR” rule	0.04|0.95	0.14|0.86	0.28|0.73	**0.06|0.96**	**0.21|0.86**	**0.06|0.97**	**0.09|0.93**	**0.09|0.95**	**0.04|0.97**	**0.04|0.97**	**0.05|0.97**	0.04|0.96	**0.17|0.88**

Cells with bold font correspond to experiments with statistically significant reconstruction of regulatory networks. See **Table 11** for abbreviations of row labels. See **Table S1** part A for a colored version of this table.

**Table 4:** Euclidean distance from the optimal algorithm with sensitivity  = 1 and specificity  = 1.

			Observational (Biological Replicate)		Observational (Environment/Time)				Semi-Experimental (Compendium)		Experimental					Method Average
Statistics	Conditioning	Post-Processing	Holstege1	Holstege2	Gresham	Gasch	Smith	Yeung	M3D	GPL90	Hughes1	Hughes2	Hu	Holstege3	Holstege4	
Fisher's Z	None	FDR, “AND” rule	0.73	**0.8**	**0.7**	**0.69**	**0.76**	0.85	0.78	**0.78**	**0.67**	**0.7**	0.74	0.79	**0.73**	**0.75**
Fisher's Z	None	FDR, “OR” rule	0.74	**0.82**	**0.7**	**0.69**	**0.78**	0.85	0.79	**0.79**	**0.67**	**0.69**	0.73	0.8	**0.74**	**0.75**
Fisher's Z	None	—	0.75	**0.82**	**0.72**	**0.7**	**0.79**	0.86	0.79	**0.8**	**0.66**	**0.66**	0.71	0.81	**0.76**	**0.76**
Fisher's Z	1 gene	“AND” rule	0.94	**0.94**	0.97	**0.93**	**0.9**	**0.9**	**0.85**	**0.81**	**0.89**	**0.89**	0.94	0.89	**0.87**	**0.90**
Fisher's Z	1 gene	“OR” rule	**0.93**	**0.93**	0.96	**0.93**	**0.89**	**0.89**	**0.85**	**0.81**	**0.89**	**0.89**	0.93	0.88	**0.86**	**0.90**
Fisher's Z	2 genes	“AND” rule	0.99	0.99	**0.99**	**0.98**	**0.97**	**0.97**	**0.97**	**0.94**	**0.97**	**0.96**	0.98	**0.96**	**0.97**	**0.97**
Fisher's Z	2 genes	“OR” rule	0.98	**0.98**	**0.98**	**0.97**	**0.96**	**0.95**	**0.95**	**0.93**	**0.96**	**0.95**	**0.97**	**0.95**	**0.95**	**0.96**
Fisher's Z	3 genes	“AND” rule	0.99	0.99	1	**0.99**	**0.97**	**0.98**	**0.99**	**0.97**	**0.98**	**0.98**	**0.99**	**0.98**	**0.98**	**0.98**
Fisher's Z	3 genes	“OR” rule	**0.98**	0.99	0.99	**0.98**	**0.96**	**0.96**	**0.96**	**0.95**	**0.97**	**0.96**	**0.98**	**0.96**	**0.96**	**0.97**
G2	None	FDR, “AND” rule	0.73	0.73	0.72	**0.7**	**0.72**	0.89	**0.89**	**0.85**	**0.78**	**0.72**	0.82	0.77	**0.71**	**0.77**
G2	None	FDR, “OR” rule	0.72	0.75	0.72	**0.7**	**0.75**	0.89	**0.9**	**0.86**	**0.74**	**0.68**	0.78	0.78	**0.73**	**0.77**
G2	None	—	0.72	0.76	0.74	**0.7**	**0.78**	0.9	**0.9**	**0.86**	**0.67**	**0.64**	0.74	0.8	**0.75**	**0.77**
G2	1 gene	“AND” rule	0.96	0.87	0.74	**0.96**	**0.82**	**0.78**	**0.71**	**0.66**	**0.98**	**0.98**	**0.96**	0.87	**0.86**	**0.86**
G2	1 gene	“OR” rule	0.96	0.86	0.74	**0.94**	**0.79**	**0.77**	**0.7**	**0.66**	**0.97**	**0.96**	**0.95**	0.86	**0.84**	**0.85**
G2	2 genes	“AND” rule	0.96	0.87	0.71	**0.96**	**0.82**	**0.96**	**0.94**	**0.86**	**0.98**	**0.98**	**0.96**	0.98	**0.86**	**0.91**
G2	2 genes	“OR” rule	0.96	0.86	0.73	**0.94**	**0.79**	**0.94**	**0.91**	**0.84**	**0.97**	**0.96**	**0.95**	0.96	**0.84**	**0.90**
G2	3 genes	“AND” rule	0.96	0.92	0.91	**0.97**	**0.84**	**0.96**	**0.94**	**0.94**	**0.98**	**0.98**	**0.96**	0.98	**0.87**	**0.94**
G2	3 genes	“OR” rule	0.96	0.87	0.77	**0.94**	**0.8**	**0.94**	**0.91**	**0.91**	**0.97**	**0.96**	**0.95**	0.96	**0.84**	**0.91**
**Dataset Average**			**0.89**	**0.88**	**0.82**	**0.87**	**0.84**	**0.90**	**0.87**	**0.85**	**0.87**	**0.86**	**0.89**	**0.89**	**0.84**	
**Data type Average**			**0.88**		**0.86**				**0.86**		**0.87**					

Cells with bold font correspond to experiments with statistically significant reconstruction of regulatory networks. See **Table 11** for abbreviations of row labels. See **Table S1** part B for a colored version of this table.

**Figure 5:**
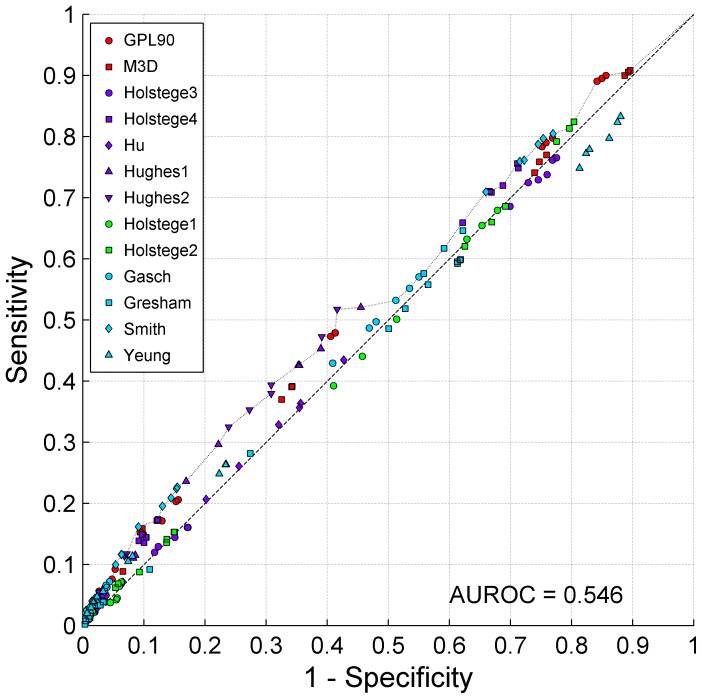
ROC curve of the Pareto frontier for sensitivity/specificity pairs obtained by application of 18 network reverse-engineering approaches to 13 datasets.

**Figure 6:**
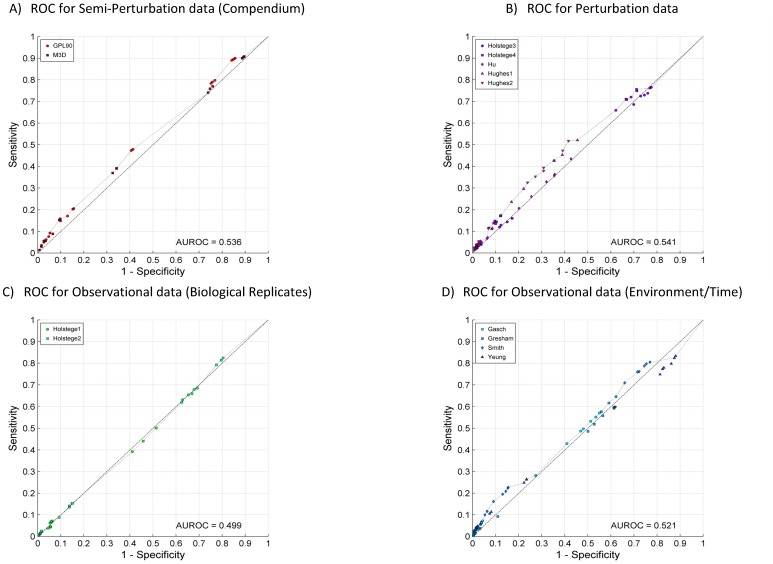
ROC curves of the Pareto frontier for sensitivity/specificity pairs obtained by application of 18 network reverse-engineering approaches to datasets of each type.

**Figure 5** provides an additional visualization of sensitivity/specificity pairs for 18 statistical approaches ×13 datasets and the corresponding ROC curve [25], [26] of the Pareto frontier [27]. The resulting area under ROC curve (AUROC) is 0.546 (p-value  = 1.12×10^−7^). **Figure 6** shows ROC curves and reports AUROC for each data type separately. It follows that observational data consisting of biological wild-type replicates leads to least accurate networks with AUROC consistent with prediction by chance (AUROC  = 0.499, p-value  = 0.55). Other data types lead to small but statistically significant AUROC values, with the best result achieved by perturbation data (AUROC  = 0.541, p-value  = 1.73×10^−6^), followed by compendium data (AUROC  = 0.536, p-value  = 2.57×10^−5^) and observational data due to change in time/environment (AUROC  = 0.521, p-value  = 0.01).

### Assessment of the accuracy of network learning with positive and negative predictive value metrics

**Table 5** provides values of positive predictive value (PPV) and negative predictive value (NPV) and **Table 6** provides a combined PPV/NPV Euclidean distance-based metric (see Methods) for 18 statistical approaches for reverse-engineering applied to 13 datasets, resulting in 234 inferred networks (see **Table S2** for a colored version of **Table 5** **and** **Table 6**, where color denotes ranking of performances). The best result for combined PPV/NPV metric ( = 0.93, corresponding to PPV  = 0.07 and NPV  = 0.98) is achieved in the Smith dataset by application of GLL with a Z test, conditioning on 3 genes and using an AND rule. The best 5% ranking results (see **Table S2** part B) according to the combined metric (17 networks out of 234) correspond to GLL with conditioning on either 2 or 3 genes. In terms of datasets, 5 out of 17 best networks originate from Yeung, 3 from Smith, 3 from Gasch, 3 from Hughes2, and the remaining 3 originate from M3D, GPL90, and Holstege4. There is a small variability in accuracy of statistical approaches averaged over 13 datasets, and the most accurate approach is GLL with Z test, conditioning on 3 genes and using an AND rule (combined metric  = 0.96 versus 0.97–0.98 for other methods). The variability in accuracy of datasets averaged over 18 statistical approaches is even smaller, and the best results are achieved in Gasch, Smith, Yeung, and Hughes2 datasets (combined metric  = 0.97 versus 0.98 for the remaining datasets). If we perform averaging over all statistical approaches and datasets belonging to the same data type, the best accuracy is achieved by observational data due to change in time/environment (0.97), followed by other data types (0.98).

**Table 5:** Positive predictive value (PPV) and negative predictive value (NPV).

			Observational (Biological Replicates)		Observational (Environment/Time)				Semi-experimental (Compendium)		Experimental				
Statistics	Conditioning	Post-Processing	Holstege1	Holstege2	Gresham	Gasch	Smith	Yeung	M3D	GPL90	Hughes1	Hughes2	Hu	Holstege3	Holstege4
Fisher's Z	None	FDR, “AND” rule	0.02|0.98	**0.02|0.98**	**0.02|0.98**	**0.02|0.98**	**0.02|0.99**	0.02|0.98	0.02|0.98	**0.02|0.98**	**0.02|0.98**	**0.02|0.98**	0.02|0.98	0.02|0.98	**0.02|0.98**
Fisher's Z	None	FDR, “OR” rule	0.02|0.98	**0.02|0.98**	**0.02|0.98**	**0.02|0.98**	**0.02|0.99**	0.02|0.98	0.02|0.98	**0.02|0.99**	**0.02|0.98**	**0.02|0.98**	0.02|0.98	0.02|0.98	**0.02|0.98**
Fisher's Z	None	None	0.02|0.98	**0.02|0.98**	**0.02|0.98**	**0.02|0.98**	**0.02|0.99**	0.02|0.98	0.02|0.98	**0.02|0.98**	**0.02|0.98**	**0.02|0.99**	0.02|0.98	0.02|0.98	**0.02|0.98**
Fisher's Z	1 gene	“AND” rule	0.02|0.98	**0.02|0.98**	0.02|0.98	**0.03|0.98**	**0.03|0.98**	**0.02|0.98**	**0.03|0.98**	**0.02|0.98**	**0.02|0.98**	**0.03|0.98**	0.02|0.98	0.02|0.98	**0.03|0.98**
Fisher's Z	1 gene	“OR” rule	**0.02|0.98**	**0.02|0.98**	0.02|0.98	**0.03|0.98**	**0.03|0.98**	**0.02|0.98**	**0.03|0.98**	**0.02|0.98**	**0.02|0.98**	**0.03|0.98**	0.02|0.98	0.02|0.98	**0.03|0.98**
Fisher's Z	2 genes	“AND” rule	0.02|0.98	0.02|0.98	**0.03|0.98**	**0.04|0.98**	**0.05|0.98**	**0.04|0.98**	**0.03|0.98**	**0.03|0.98**	**0.03|0.98**	**0.04|0.98**	0.02|0.98	**0.03|0.98**	**0.04|0.98**
Fisher's Z	2 genes	“OR” rule	0.02|0.98	**0.03|0.98**	**0.02|0.98**	**0.04|0.98**	**0.04|0.98**	**0.03|0.98**	**0.03|0.98**	**0.03|0.98**	**0.02|0.98**	**0.03|0.98**	**0.02|0.98**	**0.02|0.98**	**0.03|0.98**
Fisher's Z	3 genes	“AND” rule	0.02|0.98	0.03|0.98	0.01|0.98	**0.05|0.98**	**0.07|0.98**	**0.05|0.98**	**0.03|0.98**	**0.03|0.98**	**0.03|0.98**	**0.05|0.98**	**0.03|0.98**	**0.03|0.98**	**0.05|0.98**
Fisher's Z	3 genes	“OR” rule	**0.02|0.98**	0.02|0.98	0.02|0.98	**0.03|0.98**	**0.05|0.98**	**0.04|0.98**	**0.04|0.98**	**0.03|0.98**	**0.03|0.98**	**0.04|0.98**	**0.03|0.98**	**0.03|0.98**	**0.03|0.98**
G2	None	FDR, “AND” rule	0.02|0.98	0.02|0.98	0.02|0.98	**0.02|0.98**	**0.02|0.99**	0.02|0.97	**0.02|0.98**	**0.02|0.99**	**0.02|0.98**	**0.02|0.98**	0.02|0.98	0.02|0.98	**0.02|0.98**
G2	None	FDR, “OR” rule	0.02|0.98	0.02|0.98	0.02|0.98	**0.02|0.98**	**0.02|0.99**	0.02|0.98	**0.02|0.98**	**0.02|0.99**	**0.02|0.98**	**0.02|0.98**	0.02|0.98	0.02|0.98	**0.02|0.99**
G2	None	None	0.02|0.98	0.02|0.98	0.02|0.98	**0.02|0.98**	**0.02|0.99**	0.02|0.98	**0.02|0.98**	**0.02|0.99**	**0.02|0.98**	**0.02|0.99**	0.02|0.98	0.02|0.98	**0.02|0.99**
G2	1 gene	“AND” rule	0.01|0.98	0.02|0.98	0.02|0.98	**0.03|0.98**	**0.03|0.98**	**0.02|0.98**	**0.02|0.98**	**0.02|0.98**	**0.03|0.98**	**0.03|0.98**	**0.03|0.98**	0.02|0.98	**0.02|0.98**
G2	1 gene	“OR” rule	0.01|0.98	0.02|0.98	0.02|0.98	**0.03|0.98**	**0.02|0.98**	**0.02|0.98**	**0.02|0.98**	**0.02|0.98**	**0.02|0.98**	**0.03|0.98**	**0.03|0.98**	0.02|0.98	**0.02|0.98**
G2	2 genes	“AND” rule	0.01|0.98	0.02|0.98	0.02|0.98	**0.03|0.98**	**0.03|0.98**	**0.04|0.98**	**0.03|0.98**	**0.03|0.98**	**0.03|0.98**	**0.03|0.98**	**0.03|0.98**	0.02|0.98	**0.02|0.98**
G2	2 genes	“OR” rule	0.01|0.98	0.02|0.98	0.02|0.98	**0.03|0.98**	**0.02|0.98**	**0.03|0.98**	**0.02|0.98**	**0.02|0.98**	**0.02|0.98**	**0.03|0.98**	**0.03|0.98**	0.02|0.98	**0.02|0.98**
G2	3 genes	“AND” rule	0.01|0.98	0.02|0.98	0.01|0.98	**0.03|0.98**	**0.03|0.98**	**0.04|0.98**	**0.03|0.98**	**0.04|0.98**	**0.03|0.98**	**0.03|0.98**	**0.03|0.98**	0.02|0.98	**0.02|0.98**
G2	3 genes	“OR” rule	0.01|0.98	0.02|0.98	0.02|0.98	**0.03|0.98**	**0.02|0.98**	**0.03|0.98**	**0.02|0.98**	**0.03|0.98**	**0.02|0.98**	**0.03|0.98**	**0.03|0.98**	0.02|0.98	**0.02|0.98**

Cells with bold font correspond to experiments with statistically significant reconstruction of regulatory networks. See **Table 11** for abbreviations of row labels. See **Table S2** part A for a colored version of this table.

**Table 6:** Euclidean distance from the optimal algorithm with PPV  = 1 and NPV  = 1.

			Observational (Biological Replicates)		Observational (Environment/Time)				Semi-Experimental (Compendium)		Experimental					Method Average
Statistics	Conditioning	Post-Processing	Holstege1	Holstege2	Gresham	Gasch	Smith	Yeung	M3D	GPL90	Hughes1	Hughes2	Hu	Holstege3	Holstege4	
Fisher's Z	None	FDR, “AND” rule	0.98	**0.98**	**0.98**	**0.98**	**0.98**	0.98	0.98	**0.98**	**0.98**	**0.98**	0.98	0.98	**0.98**	**0.98**
Fisher's Z	None	FDR, “OR” rule	0.98	**0.98**	**0.98**	**0.98**	**0.98**	0.98	0.98	**0.98**	**0.98**	**0.98**	0.98	0.98	**0.98**	**0.98**
Fisher's Z	None	None	0.98	**0.98**	**0.98**	**0.98**	**0.98**	0.98	0.98	**0.98**	**0.98**	**0.98**	0.98	0.98	**0.98**	**0.98**
Fisher's Z	1 gene	“AND” rule	0.98	**0.98**	0.98	**0.97**	**0.97**	**0.98**	**0.97**	**0.98**	**0.98**	**0.97**	0.98	0.98	**0.97**	**0.98**
Fisher's Z	1 gene	“OR” rule	**0.98**	**0.98**	0.98	**0.97**	**0.97**	**0.98**	**0.97**	**0.98**	**0.98**	**0.97**	0.98	0.98	**0.97**	**0.98**
Fisher's Z	2 genes	“AND” rule	0.98	0.98	**0.97**	**0.96**	**0.95**	**0.96**	**0.97**	**0.97**	**0.97**	**0.96**	0.98	**0.97**	**0.97**	**0.97**
Fisher's Z	2 genes	“OR” rule	0.98	**0.97**	**0.98**	**0.96**	**0.96**	**0.97**	**0.97**	**0.97**	**0.98**	**0.97**	**0.98**	**0.98**	**0.97**	**0.97**
Fisher's Z	3 genes	“AND” rule	0.98	0.97	0.99	**0.95**	**0.93**	**0.95**	**0.97**	**0.97**	**0.97**	**0.95**	**0.97**	**0.97**	**0.95**	**0.96**
Fisher's Z	3 genes	“OR” rule	**0.98**	0.98	0.98	**0.97**	**0.96**	**0.96**	**0.96**	**0.97**	**0.97**	**0.96**	**0.97**	**0.97**	**0.97**	**0.97**
G2	None	FDR, “AND” rule	0.98	0.98	0.98	**0.98**	**0.98**	0.98	**0.98**	**0.98**	**0.98**	**0.98**	0.98	0.98	**0.98**	**0.98**
G2	None	FDR, “OR” rule	0.98	0.98	0.98	**0.98**	**0.98**	0.98	**0.98**	**0.98**	**0.98**	**0.98**	0.98	0.98	**0.98**	**0.98**
G2	None	None	0.98	0.98	0.98	**0.98**	**0.98**	0.98	**0.98**	**0.98**	**0.98**	**0.98**	0.98	0.98	**0.98**	**0.98**
G2	1 gene	“AND” rule	0.99	0.98	0.98	**0.97**	**0.97**	**0.98**	**0.98**	**0.98**	**0.97**	**0.97**	**0.97**	0.98	**0.98**	**0.98**
G2	1 gene	“OR” rule	0.99	0.98	0.98	**0.97**	**0.98**	**0.98**	**0.98**	**0.98**	**0.98**	**0.97**	**0.97**	0.98	**0.98**	**0.98**
G2	2 genes	“AND” rule	0.99	0.98	0.98	**0.97**	**0.97**	**0.96**	**0.97**	**0.97**	**0.97**	**0.97**	**0.97**	0.98	**0.98**	**0.97**
G2	2 genes	“OR” rule	0.99	0.98	0.98	**0.97**	**0.98**	**0.97**	**0.98**	**0.98**	**0.98**	**0.97**	**0.97**	0.98	**0.98**	**0.98**
G2	3 genes	“AND” rule	0.99	0.98	0.99	**0.97**	**0.97**	**0.96**	**0.97**	**0.96**	**0.97**	**0.97**	**0.97**	0.98	**0.98**	**0.97**
G2	3 genes	“OR” rule	0.99	0.98	0.98	**0.97**	**0.98**	**0.97**	**0.98**	**0.97**	**0.98**	**0.97**	**0.97**	0.98	**0.98**	**0.98**
**Dataset Average**			**0.98**	**0.98**	**0.98**	**0.97**	**0.97**	**0.97**	**0.98**	**0.98**	**0.98**	**0.97**	**0.98**	**0.98**	**0.98**	
**Data type Average**			**0.98**		**0.97**				**0.98**		**0.98**					

Cells with bold font correspond to experiments with statistically significant reconstruction of regulatory networks. See **Table 11** for abbreviations of row labels. See **Table S2** part B for a colored version of this table.

### Assessment of the accuracy of network learning with recall and precision metrics

**Table 7** provides values of recall (sensitivity) and precision (PPV) and **Table 8** provides a combined recall/precision Euclidean distance-based metric (see Methods) for 18 statistical approaches for reverse-engineering applied to 13 datasets, resulting in 234 inferred networks (see **Table S3** for a colored version of **Table 7** and **Table 8**, where color denotes ranking of performances). The best results for combined recall/precision metric ( = 0.99, corresponding to recall  = 0.89–0.91 and precision  = 0.02) are achieved in GPL90 and M3D datasets by application of bivariate analysis with G2 test. The best 5% ranking results (see **Table S3** part B) according to the combined metric (17 networks out of 234) also correspond to bivariate analysis. In terms of datasets, 5 out of 17 best networks originate from GPL90, 3 from M3D, 3 from Yeung, 3 from Smith, and 3 from Holstege2. There is a large variability in accuracy of statistical approaches averaged over 13 datasets, and the most accurate approaches are bivariate (combined metric  = 1.04–1.09 versus 1.27–1.38 for other methods). The variability in accuracy of datasets averaged over 18 statistical approaches is smaller, and the best results are achieved in GPL90 (combined metric  = 1.19), Smith (1.20), and Gresham (1.20) datasets (versus 1.21–1.31 for the remaining datasets). If we perform averaging over all statistical approaches and datasets belonging to the same data type, the best accuracy is achieved by compendium data (1.20), followed by observational data due to change in time/environment (1.23), observational data consisting of biological wild-type replicates (1.26), and perturbation data (1.27).

**Table 7:** Recall (sensitivity) and precision (PPV).

			Observational (Biological Replicates)		Observational (Environment/Time)				Semi-experimental (Compendium)		Experimental				
Statistics	Conditioning	Post-Processing	Holstege1	Holstege2	Gresham	Gasch	Smith	Yeung	M3D	GPL90	Hughes1	Hughes2	Hu	Holstege3	Holstege4
Fisher's Z	None	FDR, “AND” rule	0.63|0.02	**0.79|0.02**	**0.58|0.02**	**0.49|0.02**	**0.76|0.02**	0.75|0.02	0.74|0.02	**0.78|0.02**	**0.43|0.02**	**0.35|0.02**	0.33|0.02	0.73|0.02	**0.71|0.02**
Fisher's Z	None	FDR, “OR” rule	0.65|0.02	**0.81|0.02**	**0.62|0.02**	**0.53|0.02**	**0.80|0.02**	0.77|0.02	0.76|0.02	**0.79|0.02**	**0.45|0.02**	**0.38|0.02**	0.36|0.02	0.74|0.02	**0.72|0.02**
Fisher's Z	None	None	0.68|0.02	**0.82|0.02**	**0.65|0.02**	**0.57|0.02**	**0.81|0.02**	0.78|0.02	0.77|0.02	**0.80|0.02**	**0.52|0.02**	**0.47|0.02**	0.43|0.02	0.77|0.02	**0.75|0.02**
Fisher's Z	1 gene	“AND” rule	0.06|0.02	**0.06|0.02**	0.03|0.02	**0.07|0.03**	**0.10|0.03**	**0.11|0.02**	**0.15|0.03**	**0.20|0.02**	**0.11|0.02**	**0.11|0.03**	0.07|0.02	0.12|0.02	**0.14|0.03**
Fisher's Z	1 gene	“OR” rule	**0.07|0.02**	**0.07|0.02**	0.04|0.02	**0.07|0.03**	**0.12|0.03**	**0.11|0.02**	**0.16|0.03**	**0.21|0.02**	**0.12|0.02**	**0.12|0.03**	0.07|0.02	0.13|0.02	**0.15|0.03**
Fisher's Z	2 genes	“AND” rule	0.01|0.02	0.01|0.02	**0.01|0.03**	**0.02|0.04**	**0.03|0.05**	**0.03|0.04**	**0.03|0.03**	**0.06|0.03**	**0.03|0.03**	**0.04|0.04**	0.02|0.02	**0.04|0.03**	**0.03|0.04**
Fisher's Z	2 genes	“OR” rule	0.02|0.02	**0.02|0.03**	**0.02|0.02**	**0.03|0.04**	**0.04|0.04**	**0.05|0.03**	**0.05|0.03**	**0.08|0.03**	**0.04|0.02**	**0.05|0.03**	**0.03|0.02**	**0.05|0.02**	**0.05|0.03**
Fisher's Z	3 genes	“AND” rule	0.01|0.02	0.01|0.03	0.00|0.01	**0.01|0.05**	**0.03|0.07**	**0.02|0.05**	**0.01|0.03**	**0.03|0.03**	**0.02|0.03**	**0.02|0.05**	**0.01|0.03**	**0.02|0.03**	**0.02|0.05**
Fisher's Z	3 genes	“OR” rule	**0.02|0.02**	0.01|0.02	0.01|0.02	**0.02|0.03**	**0.04|0.05**	**0.04|0.04**	**0.04|0.04**	**0.05|0.03**	**0.03|0.03**	**0.04|0.04**	**0.02|0.03**	**0.04|0.03**	**0.04|0.03**
G2	None	FDR, “AND” rule	0.39|0.02	0.62|0.02	0.49|0.02	**0.43|0.02**	**0.71|0.02**	0.80|0.02	**0.90|0.02**	**0.89|0.02**	**0.24|0.02**	**0.32|0.02**	0.21|0.02	0.69|0.02	**0.66|0.02**
G2	None	FDR, “OR” rule	0.44|0.02	0.66|0.02	0.56|0.02	**0.50|0.02**	**0.76|0.02**	0.82|0.02	**0.91|0.02**	**0.90|0.02**	**0.30|0.02**	**0.39|0.02**	0.26|0.02	0.72|0.02	**0.71|0.02**
G2	None	None	0.50|0.02	0.69|0.02	0.60|0.02	**0.55|0.02**	**0.79|0.02**	0.83|0.02	**0.91|0.02**	**0.90|0.02**	**0.43|0.02**	**0.52|0.02**	0.36|0.02	0.76|0.02	**0.76|0.02**
G2	1 gene	“AND” rule	0.04|0.01	0.14|0.02	0.59|0.02	**0.04|0.03**	**0.19|0.03**	**0.25|0.02**	**0.37|0.02**	**0.47|0.02**	**0.02|0.03**	**0.02|0.03**	**0.04|0.03**	0.14|0.02	**0.14|0.02**
G2	1 gene	“OR” rule	0.04|0.01	0.15|0.02	0.60|0.02	**0.06|0.03**	**0.22|0.02**	**0.26|0.02**	**0.39|0.02**	**0.48|0.02**	**0.04|0.02**	**0.04|0.03**	**0.05|0.03**	0.16|0.02	**0.17|0.02**
G2	2 genes	“AND” rule	0.04|0.01	0.14|0.02	0.52|0.02	**0.04|0.03**	**0.19|0.03**	**0.04|0.04**	**0.06|0.03**	**0.15|0.03**	**0.02|0.03**	**0.02|0.03**	**0.04|0.03**	0.02|0.02	**0.14|0.02**
G2	2 genes	“OR” rule	0.04|0.01	0.15|0.02	0.60|0.02	**0.06|0.03**	**0.22|0.02**	**0.06|0.03**	**0.09|0.02**	**0.17|0.02**	**0.04|0.02**	**0.04|0.03**	**0.05|0.03**	0.04|0.02	**0.17|0.02**
G2	3 genes	“AND” rule	0.04|0.01	0.09|0.02	0.09|0.01	**0.03|0.03**	**0.16|0.03**	**0.04|0.04**	**0.06|0.03**	**0.06|0.04**	**0.02|0.03**	**0.02|0.03**	**0.04|0.03**	0.02|0.02	**0.14|0.02**
G2	3 genes	“OR” rule	0.04|0.01	0.14|0.02	0.28|0.02	**0.06|0.03**	**0.21|0.02**	**0.06|0.03**	**0.09|0.02**	**0.09|0.03**	**0.04|0.02**	**0.04|0.03**	**0.05|0.03**	0.04|0.02	**0.17|0.02**

Cells with bold font correspond to experiments with statistically significant reconstruction of regulatory networks. See **Table 11** for abbreviations of row labels. See **Table S3** part A for a colored version of this table.

**Table 8:** Euclidean distance from the optimal algorithm with Sensitivity = 1 and PPV  = 1.

			Observational (Biological Replicates)		Observational (Environment/Time)				Semi-Experimental (Compendium)		Experimental					Method Average
Statistics	Conditioning	Post-Processing	Holstege1	Holstege2	Gresham	Gasch	Smith	Yeung	M3D	GPL90	Hughes1	Hughes2	Hu	Holstege3	Holstege4	
Fisher's Z	None	FDR, “AND” rule	1.05	**1**	**1.07**	**1.11**	**1.01**	1.02	1.02	**1.01**	**1.14**	**1.17**	1.19	1.02	**1.02**	**1.06**
Fisher's Z	None	FDR, “OR” rule	1.04	**1**	**1.05**	**1.09**	**1**	1.01	1.01	**1**	**1.12**	**1.16**	1.17	1.02	**1.02**	**1.05**
Fisher's Z	None	None	1.03	**1**	**1.04**	**1.07**	**1**	1.01	1.01	**1**	**1.09**	**1.11**	1.13	1.01	**1.01**	**1.04**
Fisher's Z	1 gene	“AND” rule	1.36	**1.36**	1.38	**1.35**	**1.32**	**1.32**	**1.29**	**1.26**	**1.32**	**1.32**	1.36	1.32	**1.3**	**1.33**
Fisher's Z	1 gene	“OR” rule	**1.35**	**1.35**	1.37	**1.34**	**1.31**	**1.32**	**1.29**	**1.26**	**1.32**	**1.31**	1.35	1.31	**1.29**	**1.32**
Fisher's Z	2 genes	“AND” rule	1.39	1.39	**1.39**	**1.37**	**1.36**	**1.37**	**1.37**	**1.35**	**1.37**	**1.36**	1.39	**1.37**	**1.37**	**1.37**
Fisher's Z	2 genes	“OR” rule	1.38	**1.38**	**1.39**	**1.37**	**1.36**	**1.36**	**1.35**	**1.34**	**1.37**	**1.35**	**1.38**	**1.36**	**1.36**	**1.37**
Fisher's Z	3 genes	“AND” rule	1.39	1.39	1.41	**1.37**	**1.35**	**1.36**	**1.38**	**1.37**	**1.38**	**1.37**	**1.38**	**1.38**	**1.36**	**1.38**
Fisher's Z	3 genes	“OR” rule	**1.38**	1.39	1.39	**1.38**	**1.35**	**1.36**	**1.36**	**1.36**	**1.37**	**1.36**	**1.38**	**1.37**	**1.36**	**1.37**
G2	None	FDR, “AND” rule	1.16	1.05	1.11	**1.14**	**1.02**	1	**0.99**	**0.99**	**1.24**	**1.19**	1.26	1.03	**1.04**	**1.09**
G2	None	FDR, “OR” rule	1.13	1.04	1.08	**1.1**	**1.01**	1	**0.99**	**0.99**	**1.2**	**1.15**	1.23	1.02	**1.02**	**1.07**
G2	None	None	1.1	1.03	1.06	**1.08**	**1**	1	**0.99**	**0.99**	**1.14**	**1.09**	1.17	1.01	**1.01**	**1.05**
G2	1 gene	“AND” rule	1.38	1.3	1.06	**1.37**	**1.26**	**1.24**	**1.17**	**1.11**	**1.38**	**1.38**	**1.37**	1.3	**1.3**	**1.28**
G2	1 gene	“OR” rule	1.37	1.3	1.06	**1.36**	**1.25**	**1.23**	**1.15**	**1.11**	**1.37**	**1.36**	**1.36**	1.29	**1.28**	**1.27**
G2	2 genes	“AND” rule	1.38	1.3	1.09	**1.37**	**1.26**	**1.36**	**1.35**	**1.29**	**1.38**	**1.38**	**1.37**	1.38	**1.3**	**1.32**
G2	2 genes	“OR” rule	1.37	1.3	1.06	**1.36**	**1.25**	**1.35**	**1.34**	**1.28**	**1.37**	**1.36**	**1.36**	1.37	**1.28**	**1.31**
G2	3 genes	“AND” rule	1.38	1.34	1.34	**1.37**	**1.28**	**1.36**	**1.35**	**1.35**	**1.38**	**1.38**	**1.37**	1.38	**1.3**	**1.35**
G2	3 genes	“OR” rule	1.37	1.31	1.22	**1.36**	**1.26**	**1.35**	**1.34**	**1.33**	**1.37**	**1.36**	**1.36**	1.37	**1.28**	**1.33**
**Dataset Average**			**1.28**	**1.24**	**1.20**	**1.28**	**1.20**	**1.22**	**1.21**	**1.19**	**1.30**	**1.29**	**1.31**	**1.24**	**1.22**	
**Data type Average**			**1.26**		**1.23**				**1.20**		**1.27**					

Cells with bold font correspond to experiments with statistically significant reconstruction of regulatory networks. See **Table 11** for abbreviations of row labels. See **Table S3** part B for a colored version of this table.

### Connectivity of transcription factors is correlated with the accuracy of learning their sub-networks

Despite the overall low but statistically significant accuracies of gene network reverse-engineering in *S. cerevisiae*, some pathways or sub-networks can be learned with high accuracy from this data. For example, application of GLL method (with Fisher's Z-test and conditioning on one gene) to Yeung dataset allowed us to learn a sub-network of direct regulatory interactions of transcription factor GCN4 (containing 44 genes) with sensitivity  = 0.50, specificity  = 0.91, PPV  = 0.24, NPV  = 0.97, which is statistically significant after adjustment for multiple comparison (**Figure S7**). We hypothesize that total connectivity of transcription factors (assessed either in gold-standard or inferred networks) is correlated with the reconstruction accuracy of their sub-networks. If this hypothesis is true, the connectivity measure may be used to identify transcription factors whose sub-networks can be learned accurately by *de novo* reverse-engineering methods.

**Figure 7:**
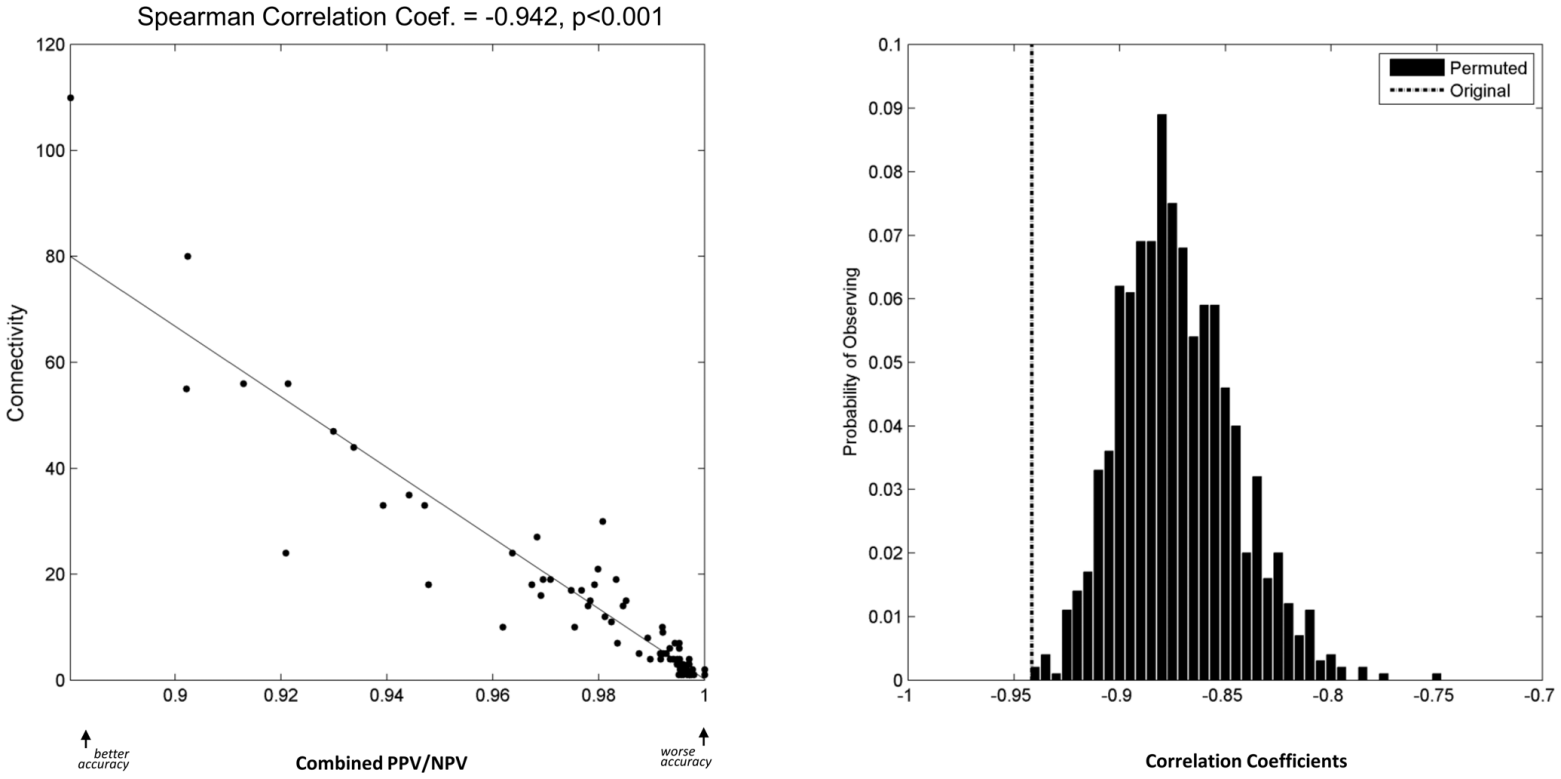
Example scatter-plot of transcription factor connectivity versus the accuracy (combined PPV/NPV metric) of reconstructing their sub-networks. The left panel shows the scatter-plot and the right panel shows the null distribution for establishing statistical significance of the observed correlation.

The left panel of **Figure 7** provides a scatter-plot showing significant correlation of transcription factor connectivity with the accuracy (combined PPV/NPV) of de novo reconstructing transcription factor sub-networks (that contain only direct regulatory interactions of each transcription factor). The right panel of **Figure 7** shows the null distribution for assessing statistical significance of this correlation. **Table 9** reports for each reverse-engineering approach and accuracy metric, the number of networks (in total we have 13 networks that were derived from 13 microarray gene expression datasets) with statistically significant correlation between connectivity of transcription factors and accuracy of reconstructing their sub-networks. As can be seen, for most reverse-engineering methods and accuracy metrics, connectivity of transcription factors in the inferred networks is significantly correlated with the reconstruction accuracy of their sub-networks. The correlations are sometimes robust and hold in multiple networks inferred from various datasets. However, the transcription factor connectivity assessed in the gold-standard networks correlates less robustly with the accuracy metrics; especially the combined sensitivity/specificity is rarely correlated. Overall, the correlations are typically negative, which implies that reverse-engineering methods can achieve higher accuracy (using each of the three combined distance metrics) for transcription factors with larger connectivity (i.e., more direct regulatory interactions). This behavior is particularly interesting for the combined sensitivity/specificity metric which is not influenced by the density of the network.

**Table 9:** Number of networks that have significant correlations between transcription factor connectivity and accuracy of reconstructing their sub-networks.

	Gold-Standard Network			Inferred Network		
Approach	Sensitivity/Specificity	PPV/NPV	Sensitivity/PPV	Sensitivity/Specificity	PPV/NPV	Sensitivity/PPV
*BIVARIATE_Z_FDR_AND*	1	1	1	8	1	10
*BIVARIATE_Z_FDR_OR*	1	0	1	9	1	11
*BIVARIATE_Z_ALPHA*	1	0	2	9	1	8
*GLL_Z_1_AND*	0	3	0	6	0	2
*GLL_Z_1_OR*	0	4	0	5	0	1
*GLL_Z_2_AND*	0	5	3	13	5	1
*GLL_Z_2_OR*	0	7	0	13	5	1
*GLL_Z_3_AND*	0	0	2	13	2	0
*GLL_Z_3_OR*	0	7	1	13	3	1
*BIVARIATE_G_FDR_AND*	0	0	1	5	3	11
*BIVARIATE_G_FDR_OR*	0	0	1	7	3	10
*BIVARIATE_G_ALPHA*	0	1	1	7	3	9
*GLL_G_1_AND*	0	1	0	4	5	10
*GLL_G_1_OR*	0	2	0	4	1	8
*GLL_G_2_AND*	0	2	0	6	6	8
*GLL_G_2_OR*	0	3	0	5	1	5
*GLL_G_3_AND*	0	2	0	7	5	8
*GLL_G_3_OR*	0	3	0	5	1	5

The correlations were assessed for 13 different networks (derived from 13 gene expression microarray datasets) for each combination of network reverse-engineering approaches and combined accuracy metrics. Statistical significance is assessed at 5% alpha level adjusted globally for multiple comparisons (over all statistical tests performed for the table). The left portion of the table corresponds to transcription factor connectivity assessed in the gold-standard network, and the right portion corresponds to transcription factor connectivity assessed in the inferred network.

## Methods and Materials

### Construction of the gold-standard networks of direct gene regulatory interactions

The general process for construction of gold-standard networks with direct gene regulatory interactions is illustrated in **Figure 1**. Two types of genome-scale data are required for network construction: (i) targeted perturbation data with gene knocks-outs/deletions or over-expressions that can be obtained by techniques for interference with RNA such as shRNA/siRNA or inducible promoters, and (ii) binding data that can be obtained by chromatin immunoprecipitation (ChIP) methods such as ChIP-chip/ChIP-seq. Targeted perturbation data allows identification of regulatory targets, while binding data allows identification of binding targets of transcription factors. Using either data alone is not sufficient to infer direct regulatory relations because regulatory interactions resulting from targeted perturbation data may be either direct or indirect, and likewise binding interactions can be either functional or not [28]. Therefore, we integrated regulatory and binding targets to obtain the set of direct regulatory targets which are graphically represented in gene regulatory networks.

In the current study, we used targeted perturbation data obtained by a co-author of this study (P.K.). The targeted perturbation data was obtained from 1,484 gene deletion (mutant) experiments. Full details of experimental procedures, normalization procedures and statistical analyses are described in [29]. In summary, mutants from independent cultures were analyzed on dual-channel 70-mer oligonucleotide arrays using a batch of wild-type RNA as a common reference. In addition, wild-type profiles were obtained to statistically assess differences with mutant profiles. All gene expression profiles were normalized by loess method [30] followed by gene-specific dye-bias correction [31]. Differentially expressed genes between wild-type and mutant profiles were determined using limma [32] at 5% alpha level adjusted for multiple comparisons using the methodology of [23], [24].

For the binding data, we used a previously published ChIP-chip dataset characterizing binding activity of 203 transcription factors to genes [33]. The original study [33] suggested using two thresholds (0.001 and 0.005) for assessing significance of binding interactions. To further filter false-positive binding relations, the study [33] suggested assessing evolutionary conservation of binding sequences in 0, 1, or 2 of the related *Saccharomyces* species. The primary approach used in the current study for identification of binding relations is based on the most conservative analysis of the above ChIP-chip data with binding threshold  = 0.001 and conservation in 2 species (resulting in “binding network #1”). In addition, we report in Supporting Information results for two other approaches: binding threshold  = 0.005 and conservation in 1 species (resulting in “binding network #2”) and binding threshold  = 0.005 without conservation requirement (resulting in “binding network #3”).

Finally, before the identified regulatory and binding relations were overlapped, all gene names were converted to systematic gene names using Saccharomyces Genome Database [34]. Any gene that has no mapping or ambiguous mapping to a systematic name was removed. This resulted in 5,395 common genes between targeted perturbation and binding data.

### Datasets for gene network reverse-engineering

We obtained 13 datasets to be used for reverse-engineering of *S. cerevisiae* gene regulatory networks. Datasets and their characteristics are listed in **Table 10**. The datasets span over 4 data types: (i) observational data consisting of biological wild-type replicates, (ii) observational data obtained by changing time and/or environmental conditions, (iii) compendium (semi-perturbation) data, and (iv) perturbation data. Data types (i) and (ii) contain samples collected by passive observation of the system without specific interference on the levels of genes. Data type (iii) was obtained by merging data from a large number of studies available in major public microarray data repositories. Those studies were predominantly perturbations-based (with gene knock-outs/over-expressions), and therefore we refer to such compendium data as “semi-perturbation”. Data type (iv) originates from gene knock-out/over-expression experiments. Out of 13 datasets used in the study, the following two are novel and are thus described in more detail below.

**Table 10:** Datasets used for gene regulatory network reverse-engineering.

Dataset type	Dataset name	Sample size	Number of genes	Description	Source	Reference
**Observational** (Biological Wild-Type Replicates)	Holstege1	200	6,170	A collection of wild-type *S. cerevisiae* strain transcriptional profiles	ArrayExpress	[29]
					E-TABM-773	
	Holstege2	200	6,170	A collection of wild-type *S. cerevisiae* strain transcriptional profiles	ArrayExpress	[29]
					E-TABM-984	
**Observational** (Environment/Time)	Gresham	100	5,590	Environmental change induced transcription response in *S. cerevisiae*	(to be submitted to GEO)	[35]
	Gasch	173	6,152	Environmental change induced transcription response in *S. cerevisiae*	http://genome-www.stanford.edu/yeast_stress/data.shtml Accessed 8/20/2014	[55]
	Smith	220	6,257	Environmental change induced transcription response in *S. cerevisiae*	GEO	[56]
					GSE9376	
	Yeung	582	5,717	Time-dependent response to rapamycin in *S. cerevisiae*	ArrayExpress	[57]
					E-MTAB-412	
**Semi-Perturbation** (Compendium)	M3D	530	5,520	Compendium dataset for *S. cerevisiae*	Many Microbe Microarrays Database (M3D)	[58]
					http://m3d.mssm.edu/ Accessed 8/20/2014	
	GPL90	1,470	6,740	Compendium dataset for *S. cerevisiae* utilizing all samples from GPL90 platform (Affymetrix Yeast Genome S98 Array)	GEO	Constructed for this study
					GPL90	
**Perturbation**	Hughes1	291	6,307	Transcriptional response in *S. cerevisiae* induced by promoter-shutoff strains	GEO	[59]
					GSE1404	
	Hughes2	291	6,307	Transcriptional response in *S. cerevisiae* induced by transcription factor overexpression and deletion	GEO	[60]
					GSE5499	
	Hu	269	6,429	Transcriptional responses in *S. cerevisiae* induced by transcription factor deletion	GEO	[54]
					GSE4654	
	Holstege3	464	6,170	Transcriptional response in *S. cerevisiae* induced by protein kinases and phosphatases deletion	ArrayExpress	[61]
					E-TABM-907	
	Holstege4	319	6,170	Transcriptional response in *S. cerevisiae* induced by non-essential knockouts of chromatin modifiers	ArrayExpress	[62]
					E-TABM-1074	

Dataset Gresham was obtained by a co-author of this study (D.G.), and it describes the transcriptional response of 5,590 S. cerevisiae genes to dynamic changes in environmental nitrogen. Cells in nitrogen limited chemostats were treated with an excess of nitrogen, and the transcriptional response was assessed at different time intervals after the nitrogen treatment, resulting in 100 gene expression profiles [35].

Dataset GPL90 was compiled by using all microarray chips from Affymetrix Yeast Genome S98 Array available in GEO [4]. Specifically, 1,509 chips with raw data (CEL files) were downloaded from GEO on 08/21/2013. RMA normalization [36] was performed on all samples using Matlab function affyrma. Data for 39 out of 1,509 chips could not be processed and therefore discarded. The remaining data for 1,470 chips were processed as one batch. Affymetrix probe sets were mapped to gene names by a customized Matlab script using the platform annotation table for GPL90 (available on GEO) as reference. A total number of 6,740 genes over 1,470 samples were obtained upon completion of the process described above. The resulting dataset is provided in **Spreadsheet S4**.

### Statistical methods for gene network reverse-engineering

This study uses de-novo statistical association-based approaches for network reverse-engineering [11], [12], [22]–[24] because they are state-of-the-art [19] and are most prevalent in the community. This is a very broad class of methods and it encompasses both traditional bivariate approaches (that consider only two genes/variables at a time) and multivariate approaches (that perform conditioning based on other genes/variables). For the latter methods we use causal graph-based techniques from the Generalized Local Learning (GLL) algorithmic family [11], [12]. Under fairly broad distributional assumptions, GLL provably discovers genes/variables that are direct causes and direct effects of the gene/variable of interest [11], [12], and is known to be one of the best performing methods for de novo gene network reverse-engineering [19].

When we infer gene networks in this study, we follow the “divide-and-conquer” (also known as “local-to-global”) approach whereby we first iteratively run each method to find direct upstream or downstream regulatory relations for each gene in the dataset, and then piece together the network. It may happen that the algorithm run on gene X may output that Y has a direct regulatory relation with X, however when the algorithm is run on gene Y, X does not belong to its output. We thus apply one of the two post-processing steps to piece together the network: (i) “AND” rule which implies that if the algorithm run on X outputs Y *and* if the algorithm run on Y outputs X, then X and Y have an edge in the resulting network, and (ii) “OR” rule which implies that if the algorithm run on X outputs Y *or* if the algorithm run on Y outputs X, then X and Y have an edge in the resulting network. Application of AND rule results in sparser networks, and OR rule results in denser networks.

The list of 18 approaches for network reverse-engineering is given in **Table 11**. Methods are based on two statistical association tests: Fisher's Z [37] and G^2^ [38] test. The latter test requires application to categorical data, and therefore we discretized gene expression data into ternary by standardizing it to mean 0 and standard deviation 1 and considering three categories: smaller than -1, between -1 and 1, and greater than 1.

**Table 11:** Statistical approaches used for gene regulatory network reverse-engineering.

Approach #	Algorithm	Statistic	Conditioning	Post-Processing	Abbreviation
1	Bivariate analysis	Fisher's Z	None	FDR, AND rule	*BIVARIATE_Z_FDR_AND*
2	Bivariate analysis	Fisher's Z	None	FDR, OR rule	*BIVARIATE_Z_FDR_OR*
3	Bivariate analysis	Fisher's Z	None	Alpha	*BIVARIATE_Z_ALPHA*
4	Multivariate causal graph-based (GLL)	Fisher's Z	1	AND rule	*GLL_Z_1_AND*
5	Multivariate causal graph-based (GLL)	Fisher's Z	1	OR rule	*GLL_Z_1_OR*
6	Multivariate causal graph-based (GLL)	Fisher's Z	2	AND rule	*GLL_Z_2_AND*
7	Multivariate causal graph-based (GLL)	Fisher's Z	2	OR rule	*GLL_Z_2_OR*
8	Multivariate causal graph-based (GLL)	Fisher's Z	3	AND rule	*GLL_Z_3_AND*
9	Multivariate causal graph-based (GLL)	Fisher's Z	3	OR rule	*GLL_Z_3_OR*
10	Bivariate analysis	G^2^	None	FDR, AND rule	*BIVARIATE_G_FDR_AND*
11	Bivariate analysis	G^2^	None	FDR, OR rule	*BIVARIATE_G_FDR_OR*
12	Bivariate analysis	G^2^	None	Alpha	*BIVARIATE_G_ALPHA*
13	Multivariate causal graph-based (GLL)	G^2^	1	AND rule	*GLL_G_1_AND*
14	Multivariate causal graph-based (GLL)	G^2^	1	OR rule	*GLL_G_1_OR*
15	Multivariate causal graph-based (GLL)	G^2^	2	AND rule	*GLL_G_2_AND*
16	Multivariate causal graph-based (GLL)	G^2^	2	OR rule	*GLL_G_2_OR*
17	Multivariate causal graph-based (GLL)	G^2^	3	AND rule	*GLL_G_3_AND*
18	Multivariate causal graph-based (GLL)	G^2^	3	OR rule	*GLL_G_3_OR*

“FDR” refers to thresholding associations at 5% FDR using the methodology of [23], [24]. “Alpha” refers to thresholding associations at 5% alpha. “AND” rule implies that if the algorithm run on X outputs Y *and* if the algorithm run on Y outputs X, then X and Y have an edge in the resulting network, and (ii) “OR” rule implies that if the algorithm run on X outputs Y *or* if the algorithm run on Y outputs X, then X and Y have an edge in the resulting network.

Finally, we note that all of the above approaches used in this study output undirected networks. Inference of directed networks from data remains a more challenging problem that is beyond the scope of the present study.

### Metrics to assess accuracy of gene network reverse-engineering

To assess accuracy of the network reverse-engineering, we used 4 core and 3 combined performance metrics. The core metrics used are: positive predictive value (PPV, also known as precision), negative predictive value (NPV), sensitivity (also known as recall), and specificity. PPV measures the probability that a regulatory interaction discovered by the algorithm exists in the gold-standard (i.e., the precision of the output network), while NPV measures the probability that an interaction not predicted by the algorithm does not exist in the gold-standard. Sensitivity measures the proportion of interactions in the gold-standard that are discovered by the algorithm (i.e., the completeness of the output network), whereas specificity measures the proportion of interactions absent in the gold-standard that are not predicted by the algorithm. The value of core metrics ranges from 0 to 1, with *larger* values corresponding to a more accurate algorithm.

Each of the three combined metrics was based on the two core antagonistic metrics and measured the Euclidean distance from the optimal algorithms with (PPV = 1, NPV = 1), (sensitivity  = 1, specificity  = 1), and (recall  = 1, precision  = 1): 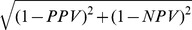, 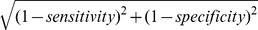, and 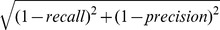, respectively. These metrics take values between 0 and 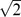, where 0 denotes performance of the optimal algorithm and 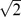 denotes performance of the worst possible algorithm. A *smaller* value for either of these two metrics implies a more accurate algorithm.

Statistical significance of the output networks was assessed using the hyper-geometric test at 5% alpha level adjusted for multiple comparisons using the methodology of [23], [24]. The adjustment was performed over 3 (gold-standards) ×18 (methods) ×13 (datasets)  = 702 applications of network reverse-engineering algorithms.

### Assessing correlation between connectivity of transcription factors and the accuracy of learning their sub-networks

For every transcription factor we measured its total connectivity (either in the inferred or gold-standard network) and accuracy of learning its sub-network measured by one of the three combined metrics mentioned in the previous subsection. Then we measured correlation using Spearman correlation coefficient and assessed significance of correlation using exact statistical test following the theory of Good [39]. The exact test is essential because transcription factors are not independent of each other. This test involved 1,000 permutations of gene identifiers for a fixed network structure and establishing a null distribution for Spearman correlation coefficients. The p-value was computed as proportion of permuted networks where correlation was higher in magnitude than the observed one. When we evaluated correlation between connectivity and accuracy for multiple networks and accuracy metrics, statistical significance was assessed at 5% alpha level adjusted for multiple comparisons using the methodology of [23], [24].

### Topological analysis and visualization of gene regulatory networks

The topological analysis of gene regulatory networks was performed in Cytoscape software platform [40] (http://www.cytoscape.org/) using NetAnalyzer plugin [41] (http://med.bioinf.mpi-inf.mpg.de/netanalyzer/). Detailed definitions and meaning of topological network parameters are given in [42]. Network visualization was performed using yED graph editor [43] (http://www.yworks.com/).

## Discussion

### Comparison with prior results

The results of the current study indicate that gene network reverse-engineering in *S. cerevisiae* is a challenging problem. Given prior work in the field, it is interesting to compare current results with the prior studies in *S. cerevisiae*, while keeping in mind that prior studies used less comprehensive gold-standard networks (see Introduction and **Table 1**). Furthermore, the majority of prior work deals only with inferring likelihood scores of all possible network edges without establishing a threshold on these scores which would result in a discrete network [18], [21]. The latter studies do not report accuracy metrics of gene *network* reverse-engineering but typically report metrics related to ranking all possible network edges by the inferred likelihood scores. To the best of our knowledge, there are only two studies which inferred discrete genome-scale networks in *S. cerevisiae*. The study [20] applied two statistical methods, resulting in non-statistically significant networks, both with PPV = 0. The study [19] used 6 versions of *S. cerevisiae* binding data-based gold-standard and applied 30 approaches (many of which were not included in the current study) to learn a network. As can be seen in **Table S7**, results of the current study are much better in terms of sensitivity and specificity and related combined metric. However, in terms of PPV, NPV, and related combined metric, results are slightly worse (by 0.01 PPV).

While this study focuses on genome-scale regulatory network reverse-engineering in *S. cerevisiae*, there was significant prior work in other model systems/organisms, e.g. *E. coli* [17]–[19], [21]. Interestingly, inference of *E. Coli* networks seems to be an easier problem than inference of *S. cerevisiae* networks. For example, the best known result in terms of combined PPV/NPV metric for *S. cerevisiae* is 0.92 (PPV = 0.08 and NPV = 0.98) but for *E. Coli* it is 0.36 (PPV = 0.64, NPV = 0.98) [19]. The results in terms of combined sensitivity/specific metric for S. cerevisiae are also worse than for E. Coli [19]. Others have also made similar observation for additional metrics [18]. It remains to be seen whether the difference in accuracy of learning S. cerevisiae and E. coli networks is due to the nature of transcription factor regulation, network complexity, quality of gold-standard networks, quality of datasets used for network learning, or combination of these factors.

### Towards improving accuracy of gene network reverse-engineering

While there are theoretical challenges of network reverse-engineering from microarray data, e.g. impact of cellular aggregation on inference of statistical relations [44], we believe that there are several ways to improve the accuracy of learning gene regulatory networks. *First*, by further improving the quality and completeness of gold-standard networks. For example, one can improve networks obtained with current approaches by ensuring that all transcription factors participate in both binding and gene knockout data and by using a large number of biological replicates for gene knockouts. The binding data can be further improved by using ChIP-seq and inclusion of other indications of bindings, for example protein binding microarrays. Another possibility worth exploring is using protein-protein interaction data in addition to binding data which would allow enriching the gold-standard networks that are currently based only on transcription factor-gene interactions. *Second*, by performing inference of gene networks from both observational and perturbation data with explicit knowledge of gene manipulations (current methods were not provided with information about targeted perturbations in the data). The latter methods (e.g., [45]–[49]) have promise because they allow to solve the theoretical problem of statistical indistinguishability of networks learned from observational data alone [6].

### More on interpretation and analysis of obtained results

We used 4 widespread core performance metrics (sensitivity or recall, specificity, PPV or precision, and NPV) and 3 ways to combine them by equally weighting two antagonistic core performance metrics at a time (sensitivity and specificity, PPV and NPV, and recall and precision). Given that most methods output sparse graphs and the underlying gold-standard networks are also sparse, the combined sensitivity/specificity metric is significantly influenced by sensitivity (because many networks have specificity ≥0.90), and in particular combined PPV/NPV metric is largely influenced by PPV (because all networks but one have NPV≥0.98). Combined recall/precision metric also suffers from similar issue since it is mostly influenced by sensitivity (because most methods have very low PPV≤0.05). The interpretation of results and relevance to specific biological problems can be improved by using other combinations of core performance metrics (e.g., by using unequal weighting of PPV and NPV metrics in the Euclidean-based combined distance metric) or by devising new performance metrics. To facilitate the latter task, we are providing in **Spreadsheet S5** detailed results with the numbers of true positive, true negative, false positive, and false negative edges computed for each network.

## Conclusions

This study has two key contributions. First, we constructed high-quality genome-scale gold-standards of direct regulatory interactions in S. cerevisiae that incorporate binding and gene knockout data. Second, we used 7 performance metrics to assess accuracy of 18 statistical association-based approaches for de-novo network reverse-engineering in 13 different real datasets spanning over 4 data types (observational data consisting of biological wild-type replicates, observational data obtained by changing time and/or environmental conditions, compendium/semi-perturbation data, and perturbation data). We found that inference of genome-scale regulatory networks in S. cerevisiae is a challenging problem and quantified resulting accuracies, most of which are statistically significant (see **Table S10**). We also found significant variability of the network reverse-engineering accuracy among statistical approaches for network inference. When accuracy is assessed based on sensitivity/specificity or recall/precision combined metrics, bivariate analysis is the best approach, and when accuracy is assessed based on PPV/NPV combined metric, Generalized Local Learning (GLL) with conditioning on 2–3 genes is the best approach. On the other hand, the variability of the network reverse-engineering accuracy is much smaller among various datasets and data types compared to variability among statistical approaches. However, some datasets/data types tend to dominate others for specific performance metrics, and in most cases using observational data consisting of biological wild-type replicates leads to worse accuracies compared with other datasets and data types. This indicates that considering that cost efficiency of various data types, observational data with changes in environments/time is preferable for network reconstruction. Finally, we found that for most reverse-engineering methods and accuracy metrics, connectivity of transcription factors is often significantly correlated with the reconstruction accuracy of their sub-networks. The correlations are sometimes robust and significant in multiple networks inferred from various datasets. Therefore, the connectivity measure may be used to identify transcription factors whose sub-networks can be learned accurately by de-novo reverse-engineering methods. We believe that the gene network reverse-engineering community will find this study useful in order to have a realistic perspective on this problem and performance of a variety of approaches.

## Supporting Information

Figure S1 **Gold-standard gene regulatory network #2.**
(PDF)

Figure S2 **Direct regulatory interactions between transcription factors in gold-standard gene regulatory network #2.**
(PDF)

Figure S3 **Topological analysis of gold-standard gene regulatory network #2.**
(PDF)

Figure S4 **Gold-standard gene regulatory network #3.**
(PDF)

Figure S5 **Direct regulatory interactions between transcription factors in gold-standard gene regulatory network #3.**
(PDF)

Figure S6 **Topological analysis of gold-standard gene regulatory network #3.**
(PDF)

Figure S7 **De-novo reconstruction of the GCN4 sub-network.**
(PDF)

Table S1 **Gold-standard network #1, sensitivity and specificity (panel A) and Euclidean distance from the optimal algorithm with sensitivity  = 1 and specificity  = 1 (panel B).**
(DOCX)

Table S2 **Gold-standard network #1, positive predictive value (PPV) and negative predictive value (NPV) (panel A) and Euclidean distance from the optimal algorithm with PPV  = 1 and NPV  = 1 (panel B).**
(DOCX)

Table S3 **Gold-standard network #1, recall (sensitivity) and precision (PPV) (panel A) and Euclidean distance from the optimal algorithm with recall  = 1 and precision  = 1 (panel B).**
(DOCX)

Table S4 **Gold-standard network #2, sensitivity and specificity (panel A) and Euclidean distance from the optimal algorithm with sensitivity  = 1 and specificity  = 1 (panel B).**
(DOCX)

Table S5 **Gold-standard network #2, positive predictive value (PPV) and negative predictive value (NPV) (panel A) and Euclidean distance from the optimal algorithm with PPV  = 1 and NPV  = 1 (panel B).**
(DOCX)

Table S6 **Gold-standard network #2, recall (sensitivity) and precision (PPV) (panel A) and Euclidean distance from the optimal algorithm with recall  = 1 and precision  = 1 (panel B).**
(DOCX)

Table S7 **Gold-standard network #3, sensitivity and specificity (panel A) and Euclidean distance from the optimal algorithm with sensitivity  = 1 and specificity  = 1 (panel B).**
(DOCX)

Table S8 **Gold-standard network #3, positive predictive value (PPV) and negative predictive value (NPV) (panel A) and Euclidean distance from the optimal algorithm with PPV  = 1 and NPV  = 1 (panel B).**
(DOCX)

Table S9 **Gold-standard network #3, recall (sensitivity) and precision (PPV) (panel A) and Euclidean distance from the optimal algorithm with recall  = 1 and precision  = 1 (panel B).**
(DOCX)

Table S10 **Comparison of accuracy of gene network reverse-engineering with the prior study.**
(DOCX)

Spreadsheet S1 **Regulatory Network.**
(XLSX)

Spreadsheet S2 **Binding Network #1.**
(XLSX)

Spreadsheet S3 **Gold-standard Network #1.**
(XLSX)

Spreadsheet S4 **GPL90 dataset.**
(XLSX)

Spreadsheet S5 **Discovery metrics for all dataset, statistical approaches and gold-standard networks.**
(XLSX)

## References

[1] Shmelkov E, Tang Z, Aifantis I, Statnikov A (2011) Assessing quality and completeness of human transcriptional regulatory pathways on a genome-wide scale. Biology Direct 6: 15. 21356087 10.1186/1745-6150-6-15 PMC3055855

[2] Huttenhower C, Hibbs MA, Myers CL, Caudy AA, Hess DC, et al (2009) The impact of incomplete knowledge on evaluation: an experimental benchmark for protein function prediction. Bioinformatics 25: 2404–2410. 19561015 10.1093/bioinformatics/btp397 PMC2735660

[3] Adriaens ME, Jaillard M, Waagmeester A, Coort SL, Pico AR, et al (2008) The public road to high-quality curated biological pathways. Drug DiscovToday 13: 856–862. 10.1016/j.drudis.2008.06.013 PMC3646620 18652912

[4] Barrett T, Troup DB, Wilhite SE, Ledoux P, Rudnev D, et al (2009) NCBI GEO: archive for high-throughput functional genomic data. Nucleic Acids Res 37: D885–D890. 18940857 10.1093/nar/gkn764 PMC2686538

[5] Parkinson H, Kapushesky M, Kolesnikov N, Rustici G, Shojatalab M, et al (2009) ArrayExpress update–from an archive of functional genomics experiments to the atlas of gene expression. Nucleic Acids Res 37: D868–D872. 19015125 10.1093/nar/gkn889 PMC2686529

[6] Spirtes P, Glymour CN, Scheines R (2000) Causation, prediction, and search. Cambridge, Mass: MIT Press.

[7] Pearl J (1988) Probabilistic reasoning in intelligent systems: networks of plausible inference. San Mateo, California: Morgan Kaufmann Publishers.

[8] Pearl J (2009) Causality: models, reasoning, and inference. Cambridge, U.K: Cambridge University Press.

[9] Glymour CN, Cooper GF (1999) Computation, causation, and discovery. Menlo Park, Calif: AAAI Press.

[10] Neapolitan RE (2004) Learning Bayesian networks. Upper Saddle River, NJ: Pearson Prentice Hall.

[11] Aliferis CF, Statnikov A, Tsamardinos I, Mani S, Koutsoukos XD (2010) Local Causal and Markov Blanket Induction for Causal Discovery and Feature Selection for Classification. Part II: Analysis and Extensions. Journal of Machine Learning Research 11: 235–284.

[12] Aliferis CF, Statnikov A, Tsamardinos I, Mani S, Koutsoukos XD (2010) Local Causal and Markov Blanket Induction for Causal Discovery and Feature Selection for Classification. Part I: Algorithms and Empirical Evaluation. Journal of Machine Learning Research 11: 171–234.

[13] Granger CWJ (1969) Investigating causal relations by econometric models and cross-spectral methods. Econometrica: Journal of the Econometric Society: 424–438.

[14] Nobelprize.org (2002) The Sveriges Riksbank Prize in Economic Sciences in Memory of Alfred Nobel 2003.

[15] Sims CA (1972) Money, income, and causality. The American Economic Review 62: 540–552.

[16] Nobelprize.org (2012) The Prize in Economic Sciences 2011.

[17] Stolovitzky G, Prill RJ, Califano A (2009) Lessons from the DREAM2 Challenges. AnnNYAcadSci 1158: 159–195. 10.1111/j.1749-6632.2009.04497.x 19348640

[18] Marbach D, Costello JC, Kuffner R, Vega NM, Prill RJ, et al (2012) Wisdom of crowds for robust gene network inference. Nature Methods 9: 796–804. 22796662 10.1038/nmeth.2016 PMC3512113

[19] Narendra V, Lytkin NI, Aliferis CF, Statnikov A (2011) A comprehensive assessment of methods for de-novo reverse-engineering of genome-scale regulatory networks. Genomics 97: 7–18. 20951196 10.1016/j.ygeno.2010.10.003 PMC3132400

[20] Bansal M, Belcastro V, Ambesi-Impiombato A, di BD (2007) How to infer gene networks from expression profiles. MolSystBiol 3: 78. 10.1038/msb4100120 PMC1828749 17299415

[21] Kuffner R, Petri T, Tavakkolkhah P, Windhager L, Zimmer R (2012) Inferring gene regulatory networks by ANOVA. Bioinformatics 28: 1376–1382. 22467911 10.1093/bioinformatics/bts143

[22] Anderson TW (2003) An introduction to multivariate statistical analysis. Hoboken, N.J: Wiley-Interscience.

[23] Benjamini Y, Yekutieli D (2001) The control of the false discovery rate in multiple testing under dependency. AnnStatist 29: 1165–1188.

[24] Benjamini Y, Hochberg Y (1995) Controlling the False Discovery Rate: A Practical and Powerful Approach to Multiple Testing. Journal of the Royal Statistical SocietySeries B (Methodological) 57: 289–300.

[25] Fawcett T (2004) ROC graphs: Notes and practical considerations for researchers. Machine Learning 31: 1–38.

[26] Fawcett T (2006) An introduction to ROC analysis. Pattern recognition letters 27: 861–874.

[27] Statnikov RB, Matusov JB (1995) Multicriteria Optimization and the Parameter Space Investigation Method. Multicriteria Optimization and Engineering: Springer. pp. 1–42.

[28] Li XY, MacArthur S, Bourgon R, Nix D, Pollard DA, et al (2008) Transcription factors bind thousands of active and inactive regions in the Drosophila blastoderm. PLoS Biol 6: e27. 18271625 10.1371/journal.pbio.0060027 PMC2235902

[29] Kemmeren P, Sameith K, Pasch LALvd, Benschop JJ, Lenstra TL, et al.. (2014) Analyzing regulatory systems by genetic perturbation of gene expression. Cell (in press).

[30] Yang YH, Dudoit S, Luu P, Lin DM, Peng V, et al (2002) Normalization for cDNA microarray data: a robust composite method addressing single and multiple slide systematic variation. Nucleic Acids Res 30: e15. 11842121 10.1093/nar/30.4.e15 PMC100354

[31] Margaritis T, Lijnzaad P, van Leenen D, Bouwmeester D, Kemmeren P, et al (2009) Adaptable gene-specific dye bias correction for two-channel DNA microarrays. Mol Syst Biol 5: 266. 19401678 10.1038/msb.2009.21 PMC2683724

[32] Smyth GK (2004) Linear models and empirical bayes methods for assessing differential expression in microarray experiments. Stat Appl Genet Mol Biol 3: 3. 10.2202/1544-6115.1027 16646809

[33] MacIsaac KD, Wang T, Gordon DB, Gifford DK, Stormo GD, et al (2006) An improved map of conserved regulatory sites for Saccharomyces cerevisiae. BMCBioinformatics 7: 113. 10.1186/1471-2105-7-113 PMC1435934 16522208

[34] Cherry JM, Hong EL, Amundsen C, Balakrishnan R, Binkley G, et al (2012) Saccharomyces Genome Database: the genomics resource of budding yeast. Nucleic Acids Res 40: D700–705. 22110037 10.1093/nar/gkr1029 PMC3245034

[35] Airoldi EM, Athanasiadou R, Brandt N, Neymotin B, Hashimoto T, et al.. (2014) Dynamics of Cell Growth and Nitrogen-regulated Gene Expression Reveals a Reciprocal Relationship between Growth and Catabolism. (Submitted).

[36] Irizarry RA, Hobbs B, Collin F, Beazer-Barclay YD, Antonellis KJ, et al (2003) Exploration, normalization, and summaries of high density oligonucleotide array probe level data. Biostatistics 4: 249–264. 12925520 10.1093/biostatistics/4.2.249

[37] Anderson TW (2003) An introduction to multivariate statistical analysis. Hoboken, N.J.: Wiley-Interscience. xx, 721 p. p.

[38] Agresti A (2002) Categorical data analysis. New York: Wiley-Interscience. xv, 710 p. p.

[39] Good PI (2000) Permutation tests: a practical guide to resampling methods for testing hypotheses. New York: Springer.

[40] Shannon P, Markiel A, Ozier O, Baliga NS, Wang JT, et al (2003) Cytoscape: a software environment for integrated models of biomolecular interaction networks. Genome Res 13: 2498–2504. 14597658 10.1101/gr.1239303 PMC403769

[41] Assenov Y, Ramirez F, Schelhorn SE, Lengauer T, Albrecht M (2008) Computing topological parameters of biological networks. Bioinformatics 24: 282–284. 18006545 10.1093/bioinformatics/btm554

[42] Doncheva NT, Assenov Y, Domingues FS, Albrecht M (2012) Topological analysis and interactive visualization of biological networks and protein structures. Nat Protoc 7: 670–685. 22422314 10.1038/nprot.2012.004

[43] Wiese R, Eiglsperger M, Kaufmann M (2004) yfiles—visualization and automatic layout of graphs. Graph Drawing Software: Springer. pp. 173–191.

[44] Chu T, Glymour C, Scheines R, Spirtes P (2003) A statistical problem for inference to regulatory structure from associations of gene expression measurements with microarrays. Bioinformatics 19: 1147–1152. 12801876 10.1093/bioinformatics/btg011

[45] Cooper GF, Yoo C (1999) Causal Discovery from a Mixture of Experimental and Observational Data. Proceedings of the Fifteenth Conference Annual Conference on Uncertainty in Artificial Intelligence (UAI-99): 116–125.

[46] Yoo C, Thorsson V, Cooper GF (2002) Discovery of causal relationships in a gene-regulation pathway from a mixture of experimental and observational DNA microarray data. Proceedings of the 2002 Pacific Symposium on Biocomputing: 498–509. 10.1142/9789812799623_0046 11928502

[47] Meganck S, Leray P, Manderick B (2006) Learning Causal Bayesian Networks from Observations and Experiments: A Decision Theoretic Approach. Modeling Decisions in Artificial Intelligence, LNCS: 58–69.

[48] Hyttinen A, Eberhardt F, Hoyer PO (2012) Learning linear cyclic causal models with latent variables. Journal of Machine Learning Research 13: 3387–3439.

[49] He Y, Geng Z (2008) Active learning of causal networks with intervention experiments and optimal designs. Journal of Machine Learning Research 9: 2523–2547.

[50] Gama-Castro S, Jimenez-Jacinto V, Peralta-Gil M, Santos-Zavaleta A, Penaloza-Spinola MI, et al (2008) RegulonDB (version 6.0): gene regulation model of Escherichia coli K-12 beyond transcription, active (experimental) annotated promoters and Textpresso navigation. Nucleic Acids Res 36: D120–D124. 18158297 10.1093/nar/gkm994 PMC2238961

[51] Lee TI, Rinaldi NJ, Robert F, Odom DT, Bar-Joseph Z, et al (2002) Transcriptional regulatory networks in Saccharomyces cerevisiae. Science 298: 799–804. 12399584 10.1126/science.1075090

[52] Teixeira MC, Monteiro P, Jain P, Tenreiro S, Fernandes AR, et al (2006) The YEASTRACT database: a tool for the analysis of transcription regulatory associations in Saccharomyces cerevisiae. Nucleic Acids Res 34: D446–D451. 16381908 10.1093/nar/gkj013 PMC1347376

[53] Monteiro PT, Mendes ND, Teixeira MC, d'Orey S, Tenreiro S, et al (2008) YEASTRACT-DISCOVERER: new tools to improve the analysis of transcriptional regulatory associations in Saccharomyces cerevisiae. Nucleic Acids Res 36: D132–D136. 18032429 10.1093/nar/gkm976 PMC2238916

[54] Hu Z, Killion PJ, Iyer VR (2007) Genetic reconstruction of a functional transcriptional regulatory network. Nat Genet 39: 683–687. 17417638 10.1038/ng2012

[55] Gasch AP, Spellman PT, Kao CM, Carmel-Harel O, Eisen MB, et al (2000) Genomic expression programs in the response of yeast cells to environmental changes. Mol Biol Cell 11: 4241–4257. 11102521 10.1091/mbc.11.12.4241 PMC15070

[56] Smith EN, Kruglyak L (2008) Gene-environment interaction in yeast gene expression. PLoS Biol 6: e83. 18416601 10.1371/journal.pbio.0060083 PMC2292755

[57] Yeung KY, Dombek KM, Lo K, Mittler JE, Zhu J, et al (2011) Construction of regulatory networks using expression time-series data of a genotyped population. Proc Natl Acad Sci U S A 108: 19436–19441. 22084118 10.1073/pnas.1116442108 PMC3228453

[58] Faith JJ, Driscoll ME, Fusaro VA, Cosgrove EJ, Hayete B, et al (2008) Many Microbe Microarrays Database: uniformly normalized Affymetrix compendia with structured experimental metadata. Nucleic Acids Res 36: D866–D870. 17932051 10.1093/nar/gkm815 PMC2238822

[59] Mnaimneh S, Davierwala AP, Haynes J, Moffat J, Peng WT, et al (2004) Exploration of essential gene functions via titratable promoter alleles. Cell 118: 31–44. 15242642 10.1016/j.cell.2004.06.013

[60] Chua G, Morris QD, Sopko R, Robinson MD, Ryan O, et al (2006) Identifying transcription factor functions and targets by phenotypic activation. Proc Natl Acad Sci U S A 103: 12045–12050. 16880382 10.1073/pnas.0605140103 PMC1567694

[61] van WS, Kemmeren P, Lijnzaad P, Margaritis T, Benschop JJ, et al (2010) Functional Overlap and Regulatory Links Shape Genetic Interactions between Signaling Pathways. Cell 143: 991–1004. 21145464 10.1016/j.cell.2010.11.021 PMC3073509

[62] Lenstra TL, Benschop JJ, Kim T, Schulze JM, Brabers NA, et al (2011) The specificity and topology of chromatin interaction pathways in yeast. Mol Cell 42: 536–549. 21596317 10.1016/j.molcel.2011.03.026 PMC4435841

