# Supplementary material for: De-Novo Learning of Genome-Scale Regulatory Networks in S. cerevisiae
Source: PLoS One. 2014 Sep 12;9(9):e106479. doi: 10.1371/journal.pone.0106479 (PMC4162580; doi:10.1371/journal.pone.0106479)
Supplement: Figure S3 — Topological analysis of gold-standard gene regulatory network #2. (PDF) [file pone.0106479.s003.pdf]

Figure S3: Topological analysis of gold-standard gene regulatory network #2.

a) Total Degree Distribution

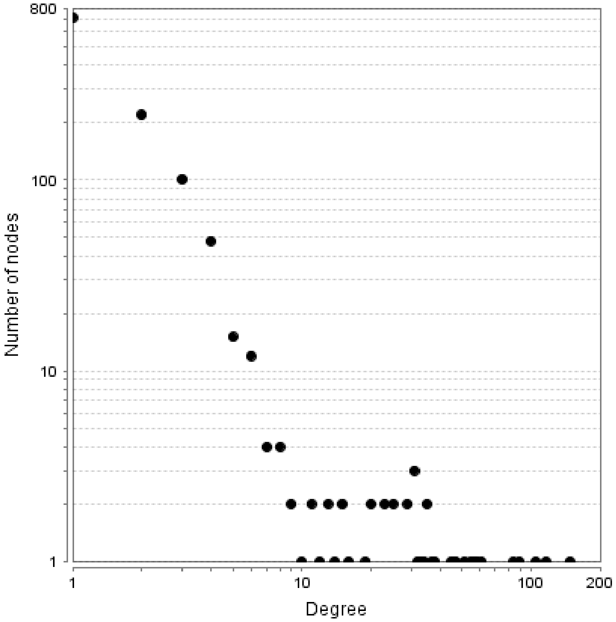

b) Shortest Path Distribution

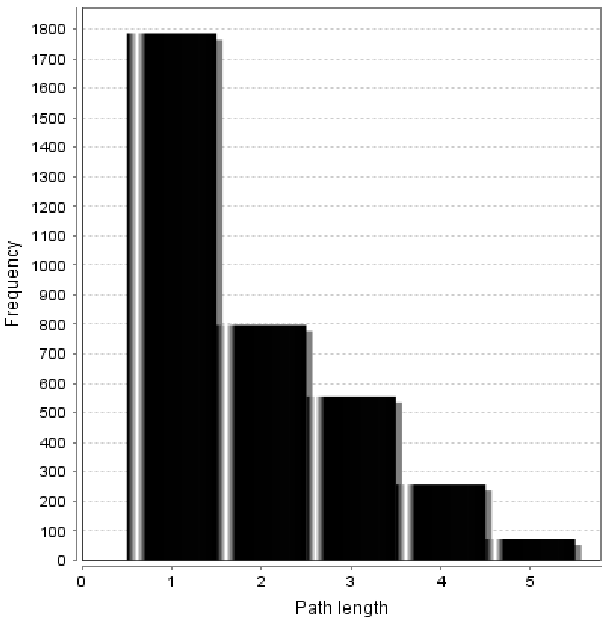

b) Average Neighborhood Connectivity Distribution

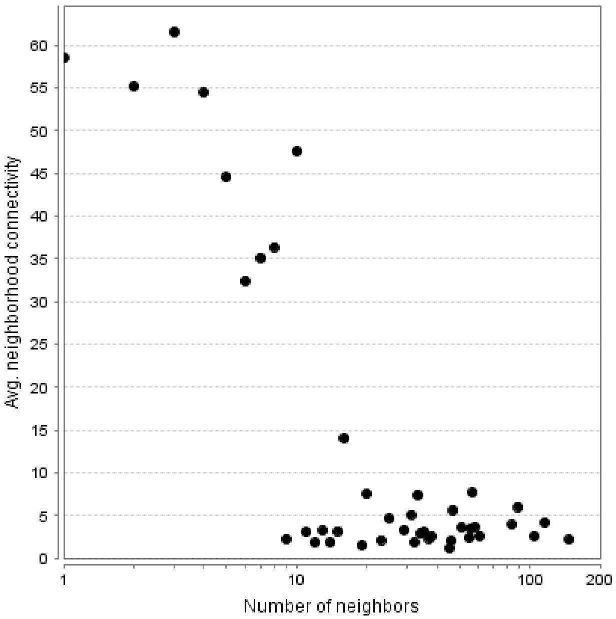

d) Network Metrics

Metric	Value
Number of Nodes	1161
Number of Edges	1785
Number of Excitatory Edges	932
Number of Inhibitory Edges	853
Network Density	0.0027
Clustering Coefficient	0.028
Number of Connected Components	7
Network Diameter	5
Network Radius	1
Shortest Paths	3467
Characteristic Path Length	1,857
Average Number of Neighbors	3.075
