# Supplementary material for: De-Novo Learning of Genome-Scale Regulatory Networks in S. cerevisiae
Source: PLoS One. 2014 Sep 12;9(9):e106479. doi: 10.1371/journal.pone.0106479 (PMC4162580; doi:10.1371/journal.pone.0106479)

**Figure S4:** Gold-standard gene regulatory network #3. Transcription factors are shown with large blue circles, and other genes are shown with small green circles. Edges in the network represent direct regulatory interactions. Inhibiting edges are shown with red, and excitatory edges are shown with black.

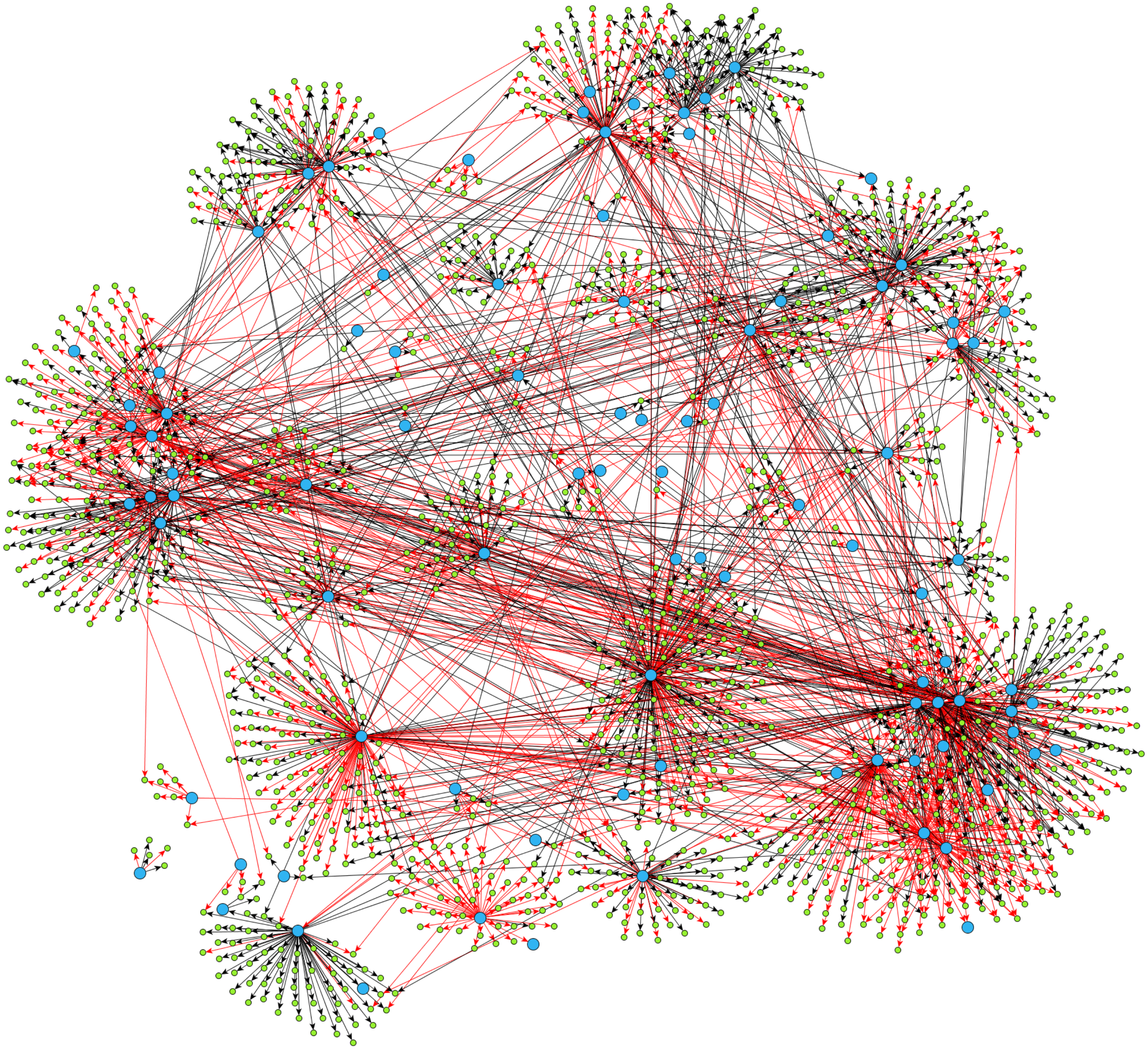

Supplement: Figure S4 — Gold-standard gene regulatory network #3. (PDF) [file pone.0106479.s004.pdf]
