# Supplementary material for: De-Novo Learning of Genome-Scale Regulatory Networks in S. cerevisiae
Source: PLoS One. 2014 Sep 12;9(9):e106479. doi: 10.1371/journal.pone.0106479 (PMC4162580; doi:10.1371/journal.pone.0106479)
Supplement: Figure S6 — Topological analysis of gold-standard gene regulatory network #3. (PDF) [file pone.0106479.s006.pdf]

Figure S6: Topological analysis of gold-standard gene regulatory network #3.

a) Total Degree Distribution

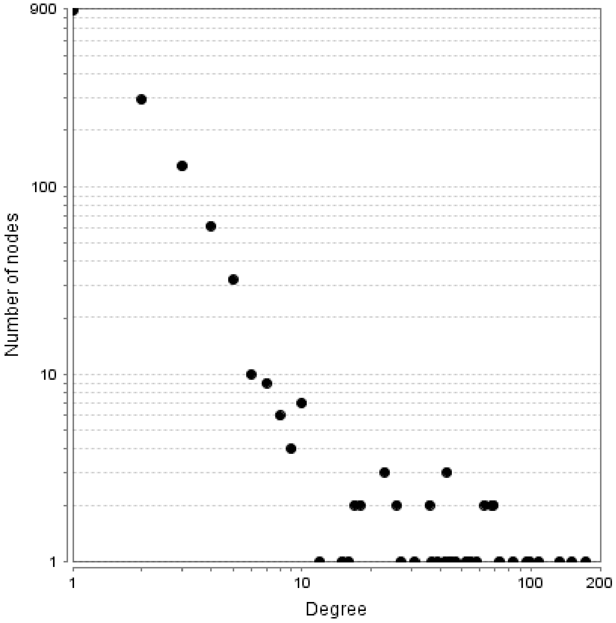

b) Shortest Path Distribution

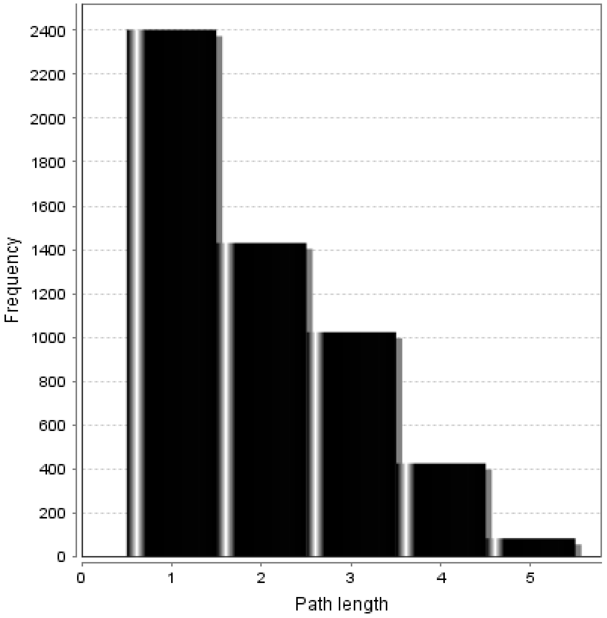

b) Average Neighborhood Connectivity Distribution

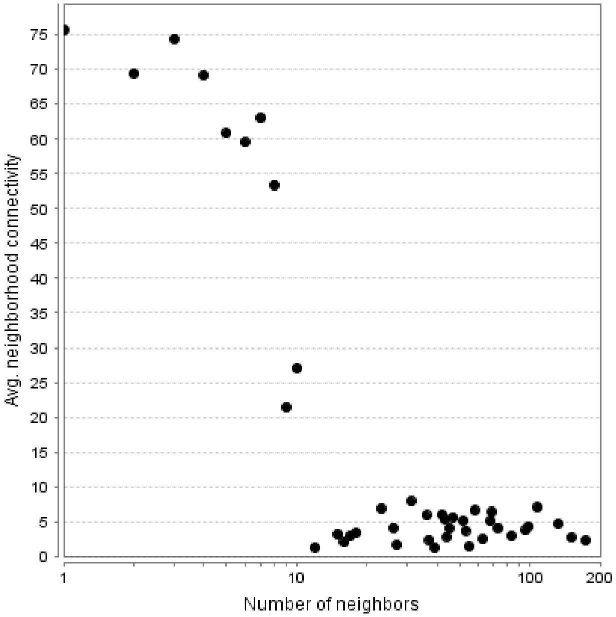

d) Network Metrics

Metric	Value
Number of Nodes	1464
Number of Edges	2403
Number of Excitatory Edges	1227
Number of Inhibitory Edges	1176
Network Density	0.0022
Clustering Coefficient	0.026
Number of Connected Components	7
Network Diameter	5
Network Radius	1
Shortest Paths	5365
Characteristic Path Length	1,948
Average Number of Neighbors	3.283
