# Supplementary material for: De-Novo Learning of Genome-Scale Regulatory Networks in S. cerevisiae
Source: PLoS One. 2014 Sep 12;9(9):e106479. doi: 10.1371/journal.pone.0106479 (PMC4162580; doi:10.1371/journal.pone.0106479)

Figure S7: De-novo reconstruction of the GCN4 sub-network. The sub-network consists of 44 genes that have direct regulatory interactions (edges) with GCN4. Out of these 44 genes, GLL identified 22 (true positives; shown with bold green borders) and missed 22 (false negatives; shown with bold red borders). GLL also introduced 69 false positives (shown with regular black borders). Transcription factors are shown with large blue circles, and other genes are shown with small green circles. Inhibiting edges are shown with red, and excitatory edges are shown with black.

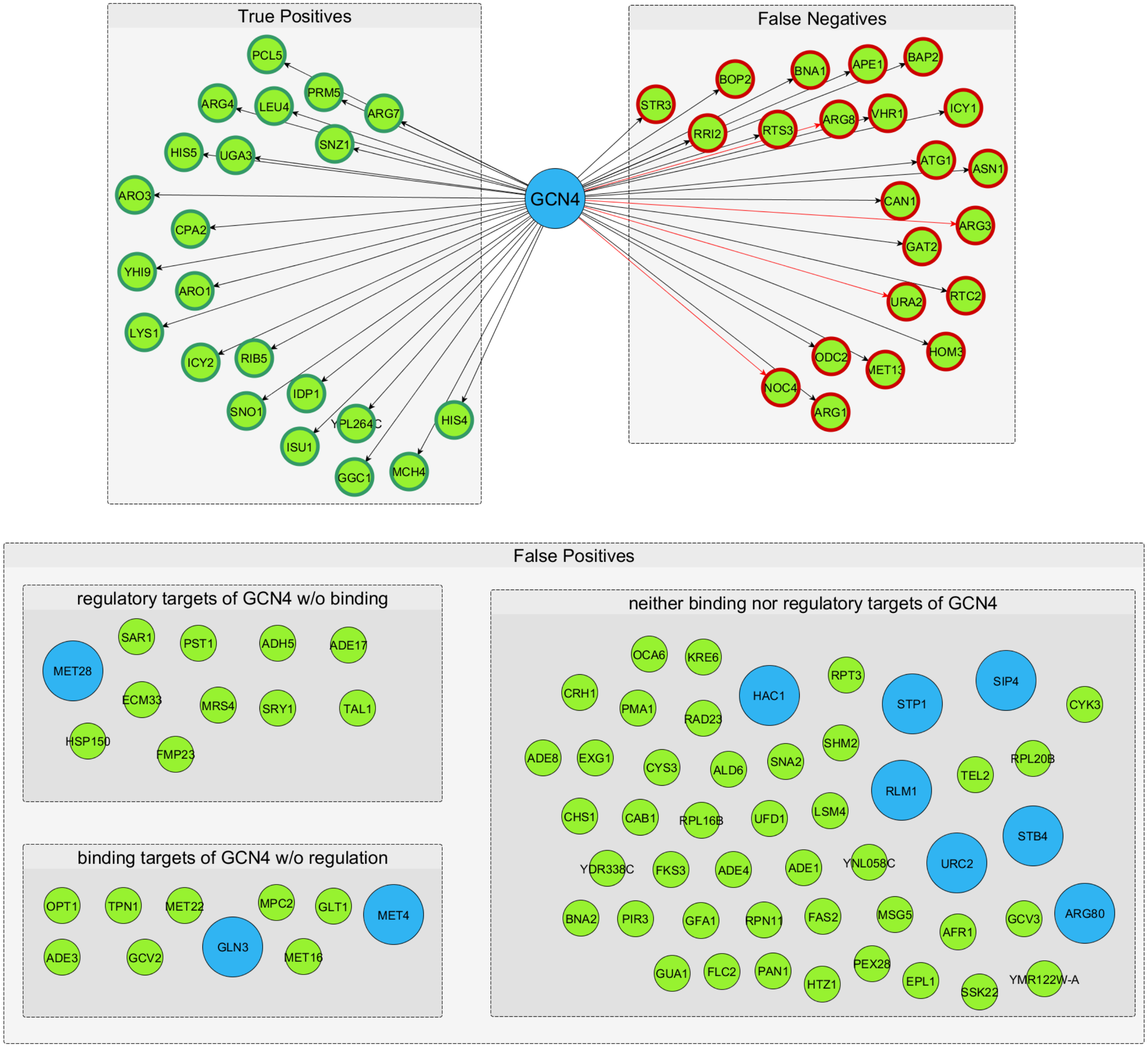

Supplement: Figure S7 — De-novo reconstruction of the GCN4 sub-network. (PDF) [file pone.0106479.s007.pdf]
