# Supplementary material for: De-Novo Learning of Genome-Scale Regulatory Networks in S. cerevisiae
Source: PLoS One. 2014 Sep 12;9(9):e106479. doi: 10.1371/journal.pone.0106479 (PMC4162580; doi:10.1371/journal.pone.0106479)
Supplement: Table S6 — Gold-standard network #2, recall (sensitivity) and precision (PPV) (panel A) and Euclidean distance from the optimal algorithm with recall = 1 and precision = 1 (panel B). (DOCX) [file pone.0106479.s013.docx]

**Table S6**: Gold-standard network #2, recall (sensitivity) and precision (PPV) (panel A) and Euclidean distance from the optimal algorithm with recall = 1 and precision = 1 (panel B). Cells with bold font correspond to experiments with statistically significant reconstruction of regulatory networks. Highlighting of cells corresponds to the accuracy of network reconstruction according to the Euclidean distance-based metric: smaller values (red) correspond to more accurate network reconstructions, and larger values (white) correspond to less accurate results. Best 5% ranking results according to the Euclidean distance-based metric are underlined in panel B. See Table 11 for abbreviations of row labels.

A)

B)
