# Supplementary material for: De-Novo Learning of Genome-Scale Regulatory Networks in S. cerevisiae
Source: PLoS One. 2014 Sep 12;9(9):e106479. doi: 10.1371/journal.pone.0106479 (PMC4162580; doi:10.1371/journal.pone.0106479)
Supplement: Table S8 — Gold-standard network #3, positive predictive value (PPV) and negative predictive value (NPV) (panel A) and Euclidean distance from the optimal algorithm with PPV = 1 and NPV = 1 (panel B). (DOCX) [file pone.0106479.s015.docx]

**Table S8**: Gold-standard network #3, positive predictive value (PPV) and negative predictive value (NPV) (panel A) and Euclidean distance from the optimal algorithm with PPV = 1 and NPV = 1 (panel B). Cells with bold font correspond to experiments with statistically significant reconstruction of regulatory networks. Highlighting of cells corresponds to the accuracy of network reconstruction according to the Euclidean distance-based metric: smaller values (red) correspond to more accurate network reconstructions, and larger values (white) correspond to less accurate results. Best 5% ranking results according to the Euclidean distance-based metric are underlined in panel B. See Table 11 for abbreviations of row labels.

A)

B)
