# Supplementary material for: De-Novo Learning of Genome-Scale Regulatory Networks in S. cerevisiae
Source: PLoS One. 2014 Sep 12;9(9):e106479. doi: 10.1371/journal.pone.0106479 (PMC4162580; doi:10.1371/journal.pone.0106479)
Supplement: Table S10 — Comparison of accuracy of gene network reverse-engineering with the prior study. (DOCX) [file pone.0106479.s017.docx]

**Table S10**: Comparison of accuracy of gene network reverse-engineering with the prior study [[1](#_ENREF_1)]. Better values of the combined Euclidean distance-based metrics are smaller (absolute best value is 0), and better values of sensitivity, specificity, PPV, and NPV are larger (absolute best value is 1).

| **Metric** | **Value in current study** | **Value in prior study** | **Performance difference*** |
| --- | --- | --- | --- |
| Combined sensitivity/specificity  Euclidean distance-based metric (best value) | 0.64  (Sensitivity = 0.52, Specificity = 0.58) | 0.75  (Sensitivity = 0.71, Specificity = 0.31) | 0.11 (c) |
| Sensitivity at specificity ≥ 0.99 | 0.04 | 0.02 | 0.02 (c) |
| Sensitivity at specificity ≥ 0.95 | 0.12 | 0.06 | 0.06 (c) |
| Sensitivity at specificity ≥ 0.90 | 0.17 | 0.11 | 0.06 (c) |
| Sensitivity at specificity ≥ 0.80 | 0.24 | 0.19 | 0.05 (c) |
| Sensitivity at specificity ≥ 0.70 | 0.39 | 0.19 | 0.20 (c) |
| Sensitivity at specificity ≥ 0.60 | 0.48 | 0.19 | 0.29 (c) |
| Combined PPV/NPV  Euclidean distance-based metric (best value) | 0.93  (PPV = 0.07,  NPV = 0.98) | 0.92  (PPV = 0.08,  NPV = 0.98) | 0.01 (p) |
| PPV at NPV ≥ 0.99 | 0.02 | 0.03 | 0.01 (p) |
| PPV at NPV ≥ 0.98 | 0.07 | 0.08 | 0.01 (p) |
| PPV at NPV ≥ 0.97 | 0.07 | 0.08 | 0.01 (p) |
| PPV at NPV ≥ 0.96 | 0.07 | 0.08 | 0.01 (p) |
| PPV at NPV ≥ 0.95 | 0.07 | 0.08 | 0.01 (p) |

* This column provides absolute value of the performance difference between current and prior studies. “(c)” means that current study has better performance; “(p)” means that prior study has better performance.

**Reference**

1. Narendra V, Lytkin NI, Aliferis CF, Statnikov A: **A comprehensive assessment of methods for de-novo reverse-engineering of genome-scale regulatory networks**. *Genomics* 2011, **97**(1):7-18.
