# Supplementary material for: De-Novo Learning of Genome-Scale Regulatory Networks in S. cerevisiae
Source: PLoS One. 2014 Sep 12;9(9):e106479. doi: 10.1371/journal.pone.0106479 (PMC4162580; doi:10.1371/journal.pone.0106479)

Figure S5: Direct regulatory interactions between transcription factors in gold-standard gene regulatory network #3. Inhibiting edges are shown with red, and excitatory edges are shown with black.

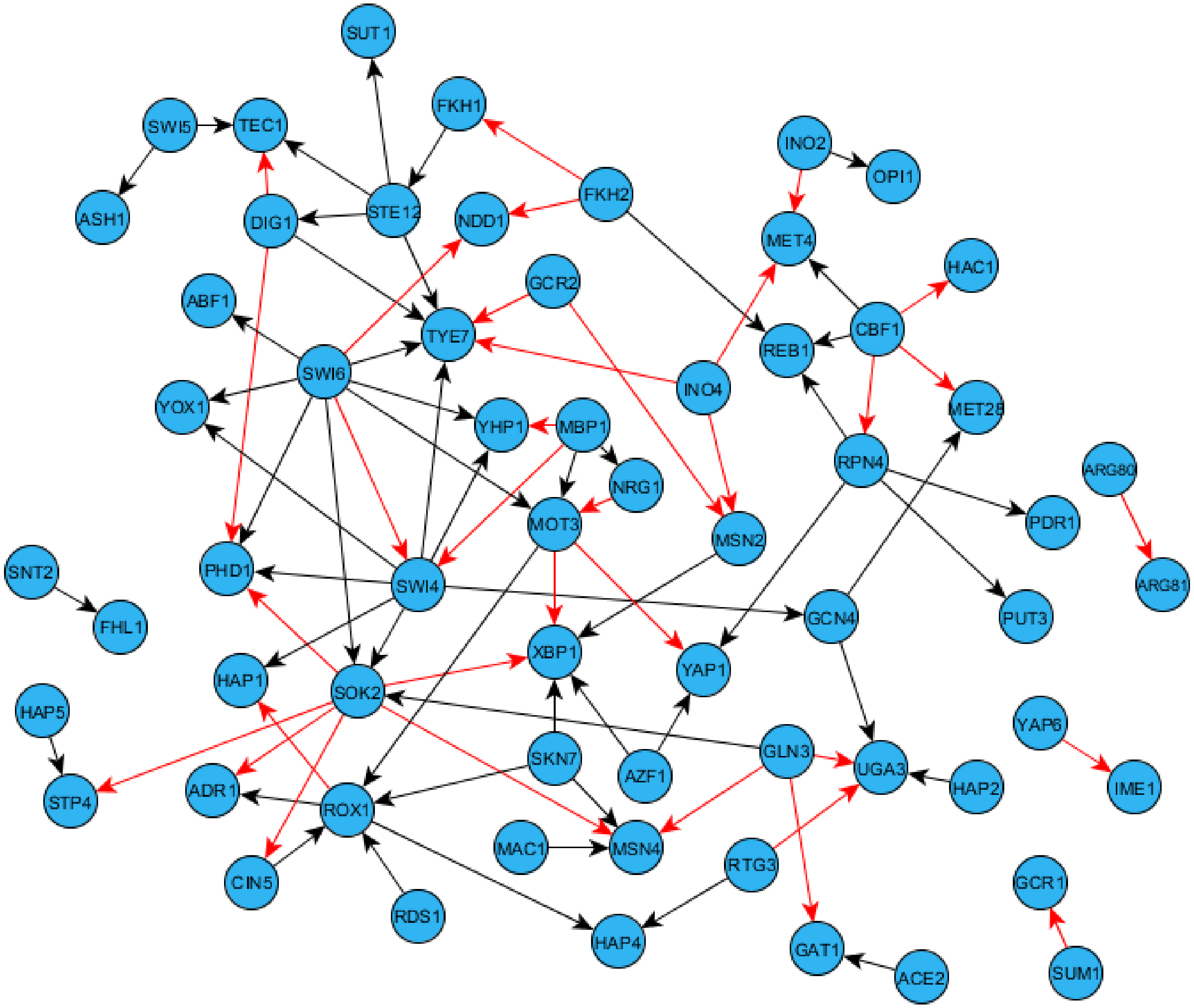

Supplement: Figure S5 — Direct regulatory interactions between transcription factors in gold-standard gene regulatory network #3. (PDF) [file pone.0106479.s005.pdf]
